# Implications of viral infections and oncogenesis in uterine cervical carcinoma etiology and pathogenesis

**DOI:** 10.3389/fmicb.2023.1194431

**Published:** 2023-05-24

**Authors:** Daming Chu, Tengteng Liu, Yuan Yao

**Affiliations:** ^1^Department of Obstetrics and Gynecology, Shengjing Hospital of China Medical University, Shenyang, China; ^2^Department of Oncology, The People’s Hospital of Liaoning Province, Shenyang, China

**Keywords:** human papillomavirus (HPV), Epstein-Barr virus (EBV), human herpesvirus (HHV), uterine cervical carcinoma (UCC), molecular mechanisms, hepatitis B and C viruses (HBV and HCV)

## Abstract

**Background:**

Uterine Cervical Carcinoma (UCC) is the most prevalent gynecological malignancy globally, with a rising incidence in recent years. Accumulating evidence indicates that specific viral infections, including human papillomavirus (HPV), Epstein-Barr virus (EBV), Hepatitis B and C viruses (HBV and HCV), and human herpesvirus (HHV), may contribute to UCC development and progression. Understanding the complex interplay between viral infections and UCC risk is crucial for developing novel preventative and therapeutic interventions.

**Methods:**

This comprehensive review investigates the association between viral infections and UCC risk by examining the roles of various viral pathogens in UCC etiology and pathogenesis, and possible molecular mechanisms. Additionally, we evaluate current diagnostic methods and potential therapeutic strategies targeting viral infections for UCC prevention or treatment.

**Results:**

The prevention of UCC has been significantly advanced by the emergence of self-sampling for HPV testing as a crucial tool, allowing for early detection and intervention. However, an essential challenge in UCC prevention lies in understanding how HPV and other viral coinfections, including EBV, HBV, HCV, HHV, HIV, or their concurrent presence, may potentially contribute to UCC development. The molecular mechanisms implicated in the association between viral infections and cervical cancer development include: (1) interference of viral oncogenes with cellular regulatory proteins, resulting in uncontrolled cell proliferation and malignant transformation; (2) inactivation of tumor suppressor genes by viral proteins; (3) evasion of host immune responses by viruses; (4) induction of a persistent inflammatory response, contributing to a tumor-promoting microenvironment; (5) epigenetic modifications that lead to aberrant gene expression; (6) stimulation of angiogenesis by viruses; and (7) activation of telomerase by viral proteins, leading to cellular immortalization. Additionally, viral coinfections can also enhance oncogenic potential through synergistic interactions between viral oncoproteins, employ immune evasion strategies, contribute to chronic inflammation, modulate host cellular signaling pathways, and induce epigenetic alterations, ultimately leading to cervical carcinogenesis.

**Conclusion:**

Recognizing the implications of viral oncogenes in UCC etiology and pathogenesis is vital for addressing the escalating burden of UCC. Developing innovative preventative and therapeutic interventions requires a thorough understanding of the intricate relationship between viral infections and UCC risk.

## Introduction

Uterine cervical carcinoma (UCC) remains a major global health concern, accounting for significant morbidity and mortality in women worldwide. Despite considerable research efforts and advances in screening and prevention strategies, UCC continues to pose a substantial public health challenge, particularly in low- and middle-income countries (Srinath et al., 2022; Ton et al., 2022; Dau et al., 2023; Tin et al., 2023). A comprehensive understanding of the etiological factors underlying the development of UCC is crucial for devising improved prevention, early detection, and treatment strategies. Although extensive research has been conducted on the etiology and pathogenesis of UCC, the precise mechanisms underlying its development are not yet fully understood. Various factors, such as age (Basoya and Anjankar, 2022; Yuan et al., 2023), obesity (Frumovitz et al., 2014; Coffey et al., 2016; Sassenou et al., 2021; Bohn et al., 2022), hormonal imbalances (Zidi et al., 2020; Iversen et al., 2021; Lasche et al., 2022), genetic predisposition (Chandra et al., 2022; Liu et al., 2022b; Yadav et al., 2023a), and environmental exposures (Kycler et al., 2017; Korsakov et al., 2022), have been implicated in UCC development. Recently, a growing body of evidence has highlighted viral infections, particularly the Human Papillomavirus (HPV), as a key factor in UCC pathogenesis (Figueiredo et al., 2023; Gilham et al., 2023; Smith et al., 2023).

Viruses, as obligate intracellular parasites, are well-recognized for their ability to manipulate host cellular processes, potentially leading to malignant transformation. Several viruses, such as HPV, Epstein-Barr virus (EBV) (Abudoukadeer et al., 2015; Luo et al., 2019), and hepatitis B and C viruses (HBV and HCV) (Mahmood et al., 2002; Ferber et al., 2003), have been identified as oncogenic, playing crucial roles in the pathogenesis of various human cancers. Nevertheless, the association between viral infections and UCC remains an active area of investigation, with inconsistent findings reported in the literature.

This review aims to provide a comprehensive overview of the current state of knowledge regarding the relationship between viral infections and UCC risk. We will discuss the evidence supporting the involvement of various viruses in the development of UCC, focusing on their potential roles in oncogenesis, molecular mechanisms, and clinical implications. Furthermore, we will explore the challenges and future directions in the study of viral infections and UCC, emphasizing the need for well-designed epidemiological and molecular studies to better understand the intricate interplay between viruses and UCC malignancy. Ultimately, elucidating the role of viral infections in UCC may lead to novel preventive and therapeutic strategies for this prevalent and life-threatening disease.

## Human papillomavirus (HPV) infection and etiology of UCC

**FIGURE 1:**
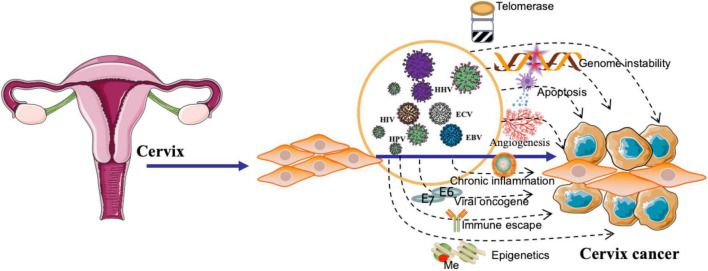
Viral pathogenesis of cervical cancer. The figure highlights the role of viral oncogenes in deregulating cellular processes, leading to uncontrolled cell proliferation and malignant transformation. Additionally, the figure emphasizes the involvement of viral infections in promoting immune escape, chronic inflammation, angiogenesis, and epigenetic modifications. Furthermore, the illustration demonstrates the influence of viruses on the activation of telomerase and the induction of genome instability, contributing to the development of cervical cancer.

There are over 100 different types of HPV, which are categorized into three risk groups: high-risk, intermediate-risk, and low-risk, based on their association with UCC (Table 1; Muñoz et al., 2003). High-risk HPV types are strongly associated with UCC, as they can cause persistent infections and lead to the development of precancerous lesions, which may eventually progress to cancer. The high-risk types include HPV strains 16, 18, 31, 33, 35, 39, 45, 51, 52, 56, 58, and 59 (Table 1). Notably, HPV 16 and 18 are the most common high-risk strains, responsible for approximately 70% of UCC cases worldwide. Two of these species, α9 (also known as HPV16-like) and α7 (also known as HPV18-like), are of particular importance in the context of UCC diagnosis (Table 1; Evans et al., 2022). HPV-16 is a high-risk HPV type belonging to the Alphapapillomavirus genus. It is responsible for nearly 50–60% of all cervical cancer cases, making it the most prevalent and oncogenic HPV type (Youn et al., 2020). The carcinogenic potential of HPV-16 is primarily attributed to the expression of two viral oncoproteins, E6 and E7. These oncoproteins play a crucial role in HPV-16-mediated cervical carcinogenesis by disrupting the normal cellular regulatory pathways. The E6 oncoprotein of HPV-16 targets the tumor suppressor protein p53, promoting its ubiquitin-mediated degradation and thus impairing its ability to regulate cell cycle progression, apoptosis, and DNA repair (Zhang et al., 2022). The E7 oncoprotein, on the other hand, binds to and inactivates the retinoblastoma protein (pRb), a key cell cycle regulator, leading to uncontrolled cell proliferation and genomic instability. Furthermore, E6 and E7 can cooperate to induce chromosomal aberrations, telomerase activation, and immortalization of infected cervical epithelial cells, eventually resulting in malignant transformation (Figure 1; Liu et al., 2019). The strong association between HPV-16 and UCC underscores the importance of HPV vaccination and screening programs to prevent infection and early detection of cervical precancerous lesions. Currently, available prophylactic HPV vaccines, such as the bivalent, quadrivalent, and nonavalent vaccines, provide protection against HPV-16 and other high-risk HPV types. These vaccination programs have been shown to significantly reduce the incidence of HPV-16-associated cervical intraepithelial neoplasia and invasive UCC (Pham et al., 2020). Moreover, persistent HPV-16 infection serves as a valuable biomarker for the early identification of women at high risk for cervical cancer development. Molecular testing for HPV-16 and other high-risk HPV types in cervical cancer screening programs can enhance the sensitivity and specificity of detecting cervical precancerous lesions, thereby improving the overall effectiveness of cervical cancer prevention strategies (Kim et al., 2020).

**TABLE 1:** Human papillomavirus (HPV) and uterine cancer risk.

Country and date	HPV strains	Main findings	Applications	References
Arbyn et al. (2022a) –United States.	HPV strains 16, 18, 31, 33, 35, 39, 45, 51, 52, 56, 58, 59, 66, 68.	Higher agreement for target amplification-based DNA assays compared to signal amplification-based DNA assays or RNA assays	HPV test agreement/concordance targets may provide criteria to extend existing validations toward alternative sampling approaches.	Arbyn et al., 2022a
Arbyn et al. (2022b)–Belgium	HPV strains 16, 18, 31, 33, 35, 39, 45, 51, 52, 56, 58, 59, 66, 68.	Compared with validated DNA assays, APTIMA HPV Test was similarly sensitive and slightly more specific for CIN2 + .	APTIMA HPV assay is a target amplification nucleic acid probe test for the *in vitro* qualitative detection of E6/E7 viral mRNA from 14 high-risk types of human papillomavirus	Arbyn et al., 2022b
Serrano et al. (2022)–Spain	HPV strains 16, 18, 31, 59, 66, 53, 33, 58, 45, 56, 52, 35, 68, 51, 39, 82, 26, 73, 6, 11, 81.	Of countries with screening programs, 12% recommend self-sampling: nine as the primary method and eight for underscreened populations.	The information can be beneficial for decision-making in both new and existing programs.	Serrano et al., 2022
Muñoz-Bello et al. (2022)–Mexico	HPV strains 16 and 18	The epidemiology of HPV-16 and HPV-18 intratype variants in the Mexican population, as well as their association with UCC.	Investigating intratype HPV variants linked to cancer may facilitate the development of targeted UCC prevention and patient outcome prediction strategies.	Muñoz-Bello et al., 2022
Evans et al. (2022)–Canada	HPV α9 species (HPV16-like), HPV α7 species (HPV18-like)	The immune landscape of HPV-positive and HPV-negative UCC displays significant disparities, with subtle differences observed between HPV α9 and α7 UCC.	Altered patient outcomes between HPV-negative and HPV-positive UCC and potentially between UCC associated with different HPV types.	Evans et al., 2022
Costa et al. (2023)–Belgium	HPV strains 16, 18, 31, 59, 66, 53, 33, 58, 45, 56, 52, 35, 68, 51, 39, 82, 26, 73, 6, 11, 81.	Opt-in strategies were less effective than send-to-all strategies.	Self-samples represent a great opportunity to increase UCC screening.	Costa et al., 2023
Schiffman et al. (2011)–United States	HPV strains 16, 18, 31, 33, 45, 52, and 58.	Discusses the role of HPV testing in UCC prevention and screening strategies	Incorporating HPV testing in UCC prevention programs could enhance screening efficacy and inform public health policy.	Schiffman et al., 2011
Walboomers et al. (1999)–Netherlands	HPV strains 16, 18, 45, 31, 33, 52, 35, and 58.	Establishes that HPV infection is a necessary cause of UCC worldwide.	The causal link between HPV infection and UCC justifies the creation and deployment of HPV vaccines to prevent UCC.	Walboomers et al., 1999
Bosch et al. (2002)–Spain	HPV strains 16, 18, 31, 33, 35, 39, 45, 51, 52, 56, 58, and 59.	Reviews the causal relationship between HPV infection and UCC.	The causal link between HPV infection and UCC justifies the creation and deployment of HPV vaccines to prevent UCC.	Bosch et al., 2002
Muñoz et al. (2003)–France, Spain	High-risk types include HPV strains 16, 18, 31, 33, 35, 39, 45, 51, 52, 56, 58, and 59. Intermediate-risk types include HPV strains 68, 73, and 82. Low-risk types include HPV strains 6, 11, 40, 42, 43, 44, 54, 61, 70, 72, 81, and CP6108.	Classifies the various HPV genotypes and their association with UCC.	HPV types associated with UCC provided a basis for the development of vaccines targeting specific high-risk HPV types.	Muñoz et al., 2003
zur Hausen (2002)–Germany	HPV strains 16, 18, 31, 33, 35, 45, 52, and 58.	Reviews the molecular mechanisms of papillomavirus in cancer development and its clinical applications.	Fundamental and clinical HPV research in cancer has contributed to the innovation of diagnostic tools, therapies, and preventive approaches.	zur Hausen, 2002
Castellsagu et al. (2001)–Spain, Mozambique	HPV strains 16, 18, 31, 33, 35, 39, 45, 51, 52, 56, 58, 59, and 66.	Investigates the HPV genotypes present in a rural population in Mozambique.	HPV genotypes in rural Mozambique informed regional vaccination programs and public health strategies tailored to specific populations.	Castellsagu et al., 2001
de Sanjose et al. (2010)–Spain, multiple international collaborators	HPV strains 16, 18, 31, 33, 35, 39, 45, 51, 52, 56, 58, 59, and 66	Analyzes the attribution of different HPV genotypes in invasive UCC cases worldwide.	HPV genotype attribution in invasive UCC helps guide the development of vaccines targeting the most common and high-risk HPV types.	de Sanjose et al., 2010
Brown et al. (2009)–United States	HPV strains 6, 11, 16, and 18	Studies the impact of the quadrivalent HPV vaccine on the infection and disease caused by non-vaccine oncogenic HPV types.	The impact of the quadrivalent HPV vaccine could be used to inform vaccination strategies and guide public health recommendations.	Brown et al., 2009
Kreimer et al. (2015)–United States, multiple international collaborators	HPV strains 6, 11, 16, 18, 31, 33, 35, 39, 45, 51, 52, 56, 58, and 59.	Evaluates the efficacy of fewer than three doses of an HPV-16/18 AS04 adjuvanted vaccine.	The efficacy of fewer than three doses of an HPV-16/18 vaccine, potentially leading to more cost-effective and accessible vaccination programs.	Kreimer et al., 2015
Joura et al. (2015)–Austria, multiple international collaborators	HPV strains 6, 11, 16, 18, 31, 33, 45, 52, and 58.	Assesses the efficacy of a valent HPV vaccine against infection and intraepithelial neoplasia in women.	The efficacy of a 9-valent HPV vaccine, potentially improving protection against a broader range of high-risk HPV types.	Joura et al., 2015
Plummer et al. (2016)–France, United Kingdom	HPV strains 16 and 18	Estimates the global burden of cancers attributable to infections, including HPV associated UCC.	The global burden of cancers attributable to infections, including HPV, informing public health policies and strategies for cancer prevention.	Plummer et al., 2016
Insinga et al. (2003)–United States	HPV strains 6 and 11	Estimates the health and economic burden of genital warts in a set of private health plans in the United States.	The health and economic burden of genital warts in the United States, highlighting the need for prevention and control strategies.	Insinga et al., 2003
Chen et al. (2018)–China		Provides an overview of cancer incidence and mortality in China, including UCC.	UCC incidence and mortality in China, informing cancer control policies and strategies, including HPV vaccination programs.	Chen et al., 2018
Tota et al. (2013)–Canada		Discusses epidemiologic approaches to evaluate the potential for HPV type replacement post vaccination.	Potential HPV type replacement post vaccination, which may guide the development and monitoring of future vaccination programs.	Tota et al., 2013
Huh et al. (2017)–United States	HPV strains 6, 11, 16, 18, 31, 33, 45, 52, and 58.	Presents the final efficacy, immunogenicity, and safety analyses of a nine-valent HPV vaccine in women aged 16-26 years.	The efficacy, immunogenicity, and safety analyses of a 9-valent HPV vaccine informed vaccination guidelines and policies.	Huh et al., 2017
Ackermann et al. (2005) Germany		UCC development because of HPV infection.	Prevention of HPV infection.	Ackermann et al., 2005
Brewster et al. (1999) United States		HPV is unrelated to the neoplastic transformation process of UCC.		Brewster et al., 1999
Montoya-Fuentes et al. (2001) Mexico	HPV strains 16, 18, 35, and 58	Viral presence in UCC and advanced squamous intraepithelial lesions is inconclusive for confirming or dismissing HPV infection involvement in HGSILs and UCC.		Montoya-Fuentes et al., 2001
Nakagawa et al. (2010) Brazil	HPV 16, 18, 31, 33, 35, 39, 45, 51, 52, 56, 58, 59, and 68.	A robust association exists between HPV infection and UCC neoplasia, which may progress to UC.	These include getting vaccinated against HPV, practicing safe sex, getting regular Pap tests, and avoiding smoking.	Nakagawa et al., 2010
Maskey et al. (2019)–Nepal		High-grade lesions exhibit greater infiltrating T cell density than low-grade lesions or normal tissue, with differences in T cell distribution between HPV-negative and HPV-positive samples.	Identifying T lymphocyte subpopulations related to cervical neoplasia grades may enable the development of targeted therapies.	Maskey et al., 2019
Sankaranarayanan et al. (2009)–India	HPV strains 16, 18, 31, 33, 35, 39, 45, 51, 52, 56, 58, 59, and 68.	Research should prioritize high-risk HPV types prevalent in rural India, with HPV16 and HPV18 demonstrating the strongest UCC risk association.	HPV testing was associated with a significant reduction in the numbers of advanced UCC and deaths from UCC.	Sankaranarayanan et al., 2009
Bhatla and Singhal (2020)–India	HPV strains 16, 18, 31, 33, 35, 39, 45, 51, 52, 56, 58, 59, 66, and 68	HPV, a necessary cause of UCC, is detected through primary HPV screening for pre-neoplastic lesions or cytology-based screening involving cell examination.	Primary HPV screening offers superior pre-neoplastic lesion detection sensitivity, improved negative test reassurance, and safely extended screening intervals.	Bhatla and Singhal, 2020
Hildesheim et al. (2001)–United States	44 HPV types (2, 6, 11, 13, 16, 18, 26, 31, 32, 33, 34, 35, 39, 40, 42, 45, 51, 52, 53, 54, 55, 56, 57, 58, 59, 61, 62, 64, 66, 67, 68, 69, 70, 72, 73, 83, AE2, AE4, AE5, AE6, AE7, AE8, W13B, AP155)	Among HPV-positive women, multiparity and smoking are risk factors for UCC. Oral contraceptive use may be associated with UCC too.	Preventing smoking and oral contraceptive use.	Hildesheim et al., 2001
Malevolti et al. (2022)–Italy		A significant association was found between cigarette smoking and the risk of UCC. The risk of CC increased with pack-years and smoking duration and decreased linearly with time since quitting, reaching that of never smokers about 15 years after quitting.	By highlighting the role of smoking in increasing UCC risk, this research supports the need for smoking cessation programs and public health policies that discourage smoking.	Malevolti et al., 2022
Marlow et al. (2007)–United Kingdom		UCC development because of HPV infection.	HPV vaccine and testing	Marlow et al., 2007
Lee et al. (2003)–Cancer letters Republic of Korea	High-risk HPV type (16/18/31/33/35/39/45/51/52/56/58/59/66/68/69) and low-risk HPV types (6/11/34/40/42/43/44).	Subjects infected with multiple HPV types had a 31.8-fold higher risk of UCC, while the single HPV type had a 19.9-fold increased risk	The detection and typing of HPV infection by HPV DNA Chip.	Lee et al., 2003
Labani et al. (2014)–India		Cervical HPV detection exhibits high sensitivity (85%) for CINIII + lesions and moderate sensitivity (53%) for CINII + lesions	HPV testing is superior to VIA and Pap test for the detection of high-grade UCC.	Labani et al., 2014
Giuliano et al. (2002)–United States	HPV strain 16 and 18	In the ≤23 years group, approximately 76% of high-risk HPV-infected individuals are potentially at risk for UCC development.	HPV testing as the sole primary screening test	Giuliano et al., 2002
Cuzick et al. (2006) United Kingdom		Among 60,000 females, HPV testing demonstrated higher CIN2 + detection sensitivity than cytology (96.1 vs. 53.0%) but lower specificity (90.7 vs. 96.3%).	HPV testing as the sole primary screening test, with cytology reserved for women who test HPV positive	Cuzick et al., 2006

Human papillomavirus α9 species includes HPV types 16, 31, 33, 35, 52, and 58. HPV α7 species includes HPV types 18, 39, 45, 59, and 68. These species comprise several high-risk HPV types, which are strongly associated with the development of UCC. UCC diagnosis typically involves a combination of screening and testing methods. Regular UCC screening, such as the Papanicolaou (Pap) test or liquid-based cytology, can identify abnormal cell changes in the cervix. If abnormal cells are detected, additional testing, such as HPV DNA testing or colposcopy, may be recommended. HPV DNA testing helps to identify high-risk HPV types, including those within the α9 and α7 species, that are associated with an increased risk of UCC. HPV α9 and α7 species, which encompass several high-risk HPV types, play a crucial role in the development of UCC. Regular screening, early detection, and appropriate follow-up care are vital for preventing the progression of precancerous lesions into invasive UCC. Additionally, HPV vaccination can help protect against the most common high-risk HPV types, including those in the α9 and α7 species, thereby reducing the risk of UCC. Intermediate-risk HPV types have a weaker association with UCC compared to high-risk types. They include HPV strains 68, 73, and 82 (Table 1). These types may contribute to the development of cancer in combination with other risk factors, but they are not as aggressive as high-risk types. Low-risk HPV types are not typically associated with UCC but can cause benign lesions, such as genital warts or mild dysplasia. These include HPV strains 6, 11, 40, 42, 43, 44, 54, 61, 70, 72, 81, and CP6108 (Table 1). Although low-risk types do not generally lead to cancer, it is still essential to monitor and treat any HPV infection to maintain overall health. The relationship between UCC and HPV types is based on the risk categories. High-risk types are the primary cause of UCC, while intermediate-risk types have a weaker association, and low-risk types are generally not linked to the development of UCC. Over time, the accumulation of genetic mutations and chromosomal abnormalities in infected cells contributes to the progression from low-grade to high-grade precancerous lesions (cervical intraepithelial neoplasia, CIN) and, eventually, to invasive UCC.

There are several proposed mechanisms by which HPV might contribute to the development of UCC, such as by promoting genomic instability (Porter and Marra, 2022), deregulating cell cycle control (Singh et al., 2022), and inhibiting apoptosis (Figure 1; Yadav et al., 2023b). HPV may downregulate the expression of major histocompatibility complex (MHC) molecules, hindering antigen presentation and recognition by cytotoxic T cells (Ferrall et al., 2021). Viral infections can induce a persistent inflammatory response, characterized by the infiltration of immune cells and the release of pro-inflammatory cytokines, chemokines, and reactive oxygen species. This chronic inflammation may contribute to the formation of a tumor-promoting microenvironment (Hemmat and Bannazadeh Baghi, 2019); HPV infection may cause epigenetic modifications, such as DNA methylation (van den Helder et al., 2022), histone modifications (Lourenço de Freitas et al., 2021), and non-coding RNA regulation (Liu et al., 2022a), which can lead to the aberrant expression of genes involved in cell cycle regulation, apoptosis, and DNA repair, thereby facilitating carcinogenesis (Figure 1). HPV can disrupt the apoptotic machinery, thereby promoting the survival and proliferation of UCC (Liu et al., 2020). HPV can cause genome instability by integrating its viral DNA into the host genome. This integration disrupts the normal function of cellular genes and regulatory elements, leading to the dysregulation of cell cycle control and DNA repair mechanisms (Figure 1; Kamal et al., 2021).

Understanding the molecular biology of HPV infection and UCC development has been crucial in developing prevention and screening strategies, such as HPV vaccines and HPV-based UCC screening tests, to reduce the incidence and mortality of UCC worldwide (Table 1). HPV vaccines, such as Gardasil (Choi et al., 2023) and Cervarix (Roy et al., 2023), protect against the most common high-risk HPV types (particularly HPV 16 and 18). By preventing infection with these types, the vaccines can effectively reduce the likelihood of developing precancerous cervical lesions and, ultimately, UCC. Widespread vaccination has the potential to substantially decrease the overall incidence of UCC. Studies have already shown a decline in the prevalence of high-risk HPV infections and precancerous cervical lesions in vaccinated populations. When a significant portion of a population is vaccinated against HPV, it can create herd immunity, which means that even unvaccinated individuals will be indirectly protected due to reduced virus circulation in the population. Herd immunity, also known as community immunity, occurs when a substantial proportion of a population is immunized against a contagious disease, subsequently reducing the overall circulation of the pathogen and indirectly protecting unvaccinated individuals (Fine et al., 2011). The impact of herd immunity in UCC prevention is particularly relevant given the etiological role of HPV in the development of this malignancy. Persistent high-risk HPV infection is responsible for virtually all cases of UCC (Bosch et al., 2002). Therefore, achieving herd immunity through widespread HPV vaccination has the potential to considerably decrease the incidence of UCC. Several factors contribute to the development of herd immunity in the context of HPV vaccination. Firstly, the widespread vaccination of adolescents, both male and female, can significantly reduce the prevalence of high-risk HPV strains in the population. This reduced prevalence can lead to a decline in the transmission of HPV to unvaccinated individuals, thereby lowering their risk of developing UCC (Markowitz et al., 2012). Secondly, herd immunity can benefit specific population groups that may be at higher risk for HPV infection or cervical cancer but have lower vaccination rates. For example, certain minority or socioeconomically disadvantaged populations might face barriers to accessing HPV vaccination. Herd immunity can provide a measure of protection to these groups by reducing the overall circulation of high-risk HPV strains (Harper and DeMars, 2017). Recent studies have provided evidence of the positive impact of herd immunity on cervical cancer prevention. A study by Drolet et al. found that in countries with high HPV vaccination coverage, the prevalence of vaccine-targeted HPV types decreased by 83% among 13–19-year-old females and 66% among 20–24-year-old females, indicating a substantial reduction in the circulation of high-risk HPV strains (Drolet et al., 2019). Furthermore, the study observed a decrease in HPV prevalence among unvaccinated females, suggesting the presence of herd immunity.

However, it is important to note that HPV vaccines do not protect against all HPV types that can cause UCC, nor do they eliminate the risk entirely. Therefore, even vaccinated individuals should continue to undergo regular UCC screenings as recommended by healthcare professionals. With a reduction in the prevalence of high-risk HPV infections and UCC, there may be a reduced need for frequent UCC screenings (e.g., Pap smears or HPV tests) and associated treatments. This can lead to lower healthcare costs and improved quality of life for women. Self-sampling for HPV testing has emerged as a valuable tool in UCC prevention, offering several benefits that can help improve the overall effectiveness of screening programs and facilitate early detection of HPV infections, particularly among high-risk groups (Racey et al., 2013; Arbyn et al., 2018). Some key advantages of self-sampling for HPV testing during UCC prevention include: Self-sampling allows women to collect their samples in the privacy of their homes, without the need for a clinical appointment. This can lead to higher participation rates, especially among women who may be reluctant or unable to attend traditional clinic-based screenings due to cultural, logistical, or financial barriers (Sancho-Garnier et al., 2013; Gupta et al., 2018). Self-sampling provides a more comfortable and less invasive alternative to clinician-collected samples. Many women find self-sampling less intimidating and more acceptable, which can encourage them to undergo regular HPV testing as part of their UCC prevention routine (Waller et al., 2009; Arrossi et al., 2016). Self-sampling can help reduce costs associated with clinic visits, clinician time, and resources (Abuelo et al., 2014). By facilitating increased participation in HPV testing, self-sampling can contribute to more cost-effective UCC screening programs (Elfström et al., 2014). Self-sampling can improve access to UCC screening among hard-to-reach populations, such as women living in remote areas or those with limited access to healthcare services (Safaeian et al., 2007). By making HPV testing more accessible, self-sampling can help reduce health disparities and improve UCC prevention efforts in underserved communities (Bennett et al., 2018). With increased participation in HPV testing through self-sampling, more women can be screened for high-risk HPV infections. Early detection of these infections allows for timely intervention, such as close monitoring or treatment, to prevent the development of precancerous lesions and UCC (Ronco et al., 2014). It is important to note that the accuracy of self-sampling depends on the quality of the sample collected and the type of HPV test used (Arbyn et al., 2014). High-quality self-sampling kits and sensitive HPV tests are essential for reliable results (Petignat et al., 2007). In conclusion, self-sampling for HPV testing is an important tool in UCC prevention, as it can increase participation rates, improve access to screening, and facilitate early detection and intervention, ultimately contributing to a reduction in the incidence of UCC (Ogilvie et al., 2007; Nelson et al., 2017). It is important to consider other risk factors for UCC, such as obesity (Urbute et al., 2022), smoking (Table 1; Hildesheim et al., 2001; Malevolti et al., 2022), hormone replacement therapy, tamoxifen use, and a family history of Lynch syndrome (Kwolek et al., 2023), as these factors have more consistent associations with UCC risk.

## The role of EBV in UCC risk

Epstein-Barr virus, also known as human herpesvirus 4 (HHV-4), is a virus that has been associated with various types of cancers, including Burkitt’s lymphoma, Hodgkin’s lymphoma, nasopharyngeal carcinoma, and some types of stomach cancer (gastric carcinoma). However, the association between EBV and UCC is not well-established. Some studies have detected EBV DNA or viral proteins in UCC tissues (Blanco et al., 2020; Castro et al., 2020; Feng M. et al., 2021; Macleod and Reynolds, 2021), while others have not found any significant association between EBV infection and UCC (Table 2; De Oliveira et al., 1999; Noel et al., 2001).

**TABLE 2:** Association of Epstein-Barr virus (EBV) with UCC risk.

Country and date	EBV strains or coinfection	Main findings	Applications	References
Blanco et al. (2020)–Chile	EBV and HPV	The study explores the epidemiological and molecular aspects of EBV and HPV coinfection, suggesting that EBV and HPV coinfection could increase the risk of UCC development by affecting multiple signaling pathways.	Investigates the role of EBV and HPV coinfection in UCC, discussing epidemiology, molecular mechanisms, and potential therapeutic targets.	Blanco et al., 2020
Castro et al. (2020)–Peru	EBV	EBNA-1 and LMP-1 in cervical cancer specimens, with the authors reporting a 22.2% prevalence in UCC patients. Tthe 1-year and 5-year OS rates were higher in the EBV-positive group compared to the EBV-negative group.	This finding highlights the potential clinical utility of EBV status as a prognostic biomarker of UCC.	Castro et al., 2020
Feng M. et al. (2021)–China	EBV, HPV, and HIV	The study reports that EBV and HPV coinfection in Chinese women living with HIV was significantly associated with high-grade cervical intraepithelial neoplasia, supporting the role of EBV as a potential cofactor in the development of cervical lesions.	Studies the role of EBV and HPV coinfection in cervical intraepithelial neoplasia in Chinese women living with HIV.	Feng M. et al., 2021
Macleod and Reynolds (2021)–Eastern and Southern Africa	EBV	This scoping review highlights the high prevalence of HPV infection and UCC among women who sell sex in Eastern and Southern Africa, emphasizing the need for targeted interventions to reduce the burden of HPV-related disease.	Reviews the prevalence of HPV infection and UCC among women who sell sex in Eastern and Southern Africa.	Macleod and Reynolds, 2021
De Oliveira et al. (1999)–Brazil	EBV	The study found no evidence of EBV infection in cervical carcinomas, suggesting that EBV may not play a significant role in UCC.	Reports the lack of EBV infection in cervical carcinomas.	De Oliveira et al., 1999
Noel et al. (2001)–Belgium	EBV	The investigation of lymphoepithelioma-like carcinoma of the uterine cervix revealed evidence of HPV infection but not EBV, indicating that HPV may be the primary causative agent.	Presents evidence of HPV infection but not EBV in lymphoepithelioma-like carcinoma of the uterine cervix.	Noel et al., 2001
Yordanov et al. (2020)–Bulgaria	EBV	The single-center study found no correlation between EBV and lymphoepithelioma-like carcinoma of the uterine cervix, while a strong association with high-risk HPV types was observed.	Examines the correlation between EBV and HPV infection in lymphoepithelioma-like carcinoma of the uterine cervix.	Yordanov et al., 2020
Kienka et al. (2019)–United States	EBV	The study reports that EBV, but not human cytomegalovirus, is associated with high-grade HPV-associated cervical lesions in women from North Carolina.	Investigates the association of EBV with high-grade HPV-associated cervical lesions in women in North Carolina.	Kienka et al., 2019
Cameron et al. (2020)–United States	EBV	HIV-infected women with genital tract specimens positive for both HPV and EBV were found to be at a higher risk for abnormal cervical cytology.	Assesses the risk of abnormal cervical cytology in HIV-infected women testing positive for both HPV and EBV.	Cameron et al., 2020
Vranic et al. (2018)–Qatar	EBV	This brief update discusses the association between EBV and UCC, highlighting the potential role of EBV as a cofactor in the development of HPV-associated cervical lesions.	Provides an update on the role of EBV in UCC.	Vranic et al., 2018
de Lima et al. (2018)–Brazil	EBV	A meta-analysis found a significant association between EBV and cervical carcinoma, supporting the role of EBV as a potential cofactor in cervical carcinogenesis.	Conducts a meta-analysis to assess the association between EBV and cervical carcinoma.	de Lima et al., 2018
Sasagawa et al. (2000)–Japan	EBV	The study found that EBV gene expression was more frequently observed in invasive UCC than in cervical intraepithelial neoplasia, suggesting a potential role for EBV in UCC progression.	Compares EBV gene expression in cervical intraepithelial neoplasia and invasive UCC, in relation to HPV infection.	Sasagawa et al., 2000
Khashman et al. (2020a)–Iraq	EBV	The study investigated the expression of EBV latent membrane protein 1 (LMP1) in Iraqi women with cervical carcinoma, although the main findings were not provided in the citation.	Examines the expression of EBV latent membrane protein 1 (LMP1) in Iraqi women with cervical carcinoma.	Khashman et al., 2020a
Yordanov et al. (2019)–Bulgaria	EBV	The immunohistochemical study showed HPV and EBV infection in patients with lymphoepithelioma-like carcinoma of the uterine cervix, supporting a possible role for both viruses in the development of this rare subtype of UCC.	Studies the immunohistochemical expression of HPV and EBV in patients with lymphoepithelioma-like carcinoma of the uterine cervix.	Yordanov et al., 2019
da Carvalho and de Melo (2019)–Brazil	HPV and EBV	The study highlights the association between HPV and EBV infections in UCC, discussing the potential interactions and molecular mechanisms involved in cancer development.	Reviews the association between HPV and EBV infections and cancer of the uterine cervix.	da Carvalho and de Melo, 2019
Lau et al. (2021)–Taiwan	HPV and EBV	The case report describes a rare occurrence of an Epstein-Barr Virus-associated smooth muscle tumor (EBV-SMT) of the cranio-cervical junction in an immunocompetent patient, emphasizing the need for further research on EBV-SMT in this population.	Presents a case history of an EBV-associated smooth muscle tumor (EBV-SMT) of the cranio-cervical junction in an immunocompetent patient.	Lau et al., 2021
Okoye et al. (2023)–Southern Nigeria	EBV-LMP1	The prevalence of HPV/EBV-LMP1 copresence was high in invasive UCC compared with non-invasive UCC cases	EBV DNA should equally be investigated during HPV testing of suspected UCC cases to identify individuals with poorer prognoses.	Okoye et al., 2023
Sosse et al. (2022)–France		The role of EBV as potential cofactors in cervical carcinogenesis.	The study could lead us to develop new therapeutics and preventive vaccines	Sosse et al., 2022

Several mechanisms have been proposed to elucidate the role of EBV in the development of UCC. Two crucial viral proteins, Epstein-Barr nuclear antigen 1 (EBNA-1) and latent membrane protein 1 (LMP-1), have been implicated in the oncogenic process during EBV infection. EBNA-1 is a multifunctional protein involved in the replication, maintenance, and segregation of the EBV episome in latently infected cells. It is expressed in all EBV-associated malignancies and plays a pivotal role in the persistence of viral episomes within host cells. Additionally, EBNA-1 contributes to the immortalization of infected cells by altering cellular gene expression and promoting genomic instability. During clinical diagnosis, the detection of EBNA-1 expression may serve as a marker of latent EBV infection and its associated cervical malignancies (Hoseini Tabatabaie et al., 2023). LMP-1, on the other hand, is a transmembrane protein that functions as a constitutively active mimic of the tumor necrosis factor receptor (TNFR) family, stimulating multiple signaling pathways that promote cell survival, proliferation, and differentiation. LMP-1 exerts its oncogenic effects by activating the nuclear factor-kappa B (NF-κB), mitogen-activated protein kinase (MAPK), and Janus kinase/signal transducer and activator of transcription (JAK/STAT) pathways. Detection of LMP-1 expression during clinical diagnosis can indicate the presence of an active EBV infection and suggest a more aggressive disease course, as LMP-1 is implicated in immune evasion, angiogenesis, and metastasis (Awasthi et al., 2023). A well-executed retrospective cohort study contributes to our understanding of the prevalence of EBV by targeting EBNA-1 and LMP-1 in cervical cancer specimens, with the authors reporting a 22.2% prevalence (*n* = 22) in their cohort of 99 patients. This is a valuable addition to the existing body of knowledge on EBV and cervical cancer. The authors provide evidence for the prognostic value of EBV status in cervical cancer patients, demonstrating that the 1-year and 5-year OS rates were higher in the EBV-positive group compared to the EBV-negative group. This finding highlights the potential clinical utility of EBV status as a prognostic biomarker (Table 2; Castro et al., 2020). EBV infection has been associated with epigenetic modifications in host cells, including DNA methylation (Fujii et al., 2022), histone modifications (Fujii et al., 2022), and non-coding RNA regulation (Kolesnik et al., 2021). EBV has developed various strategies to evade the host immune response, allowing the virus to persist in infected cells. This immune evasion can lead to chronic inflammation and contribute to the development of a favorable microenvironment for UCC initiation and progression (Figure 1; Pandey et al., 2021).

There have been some studies suggesting that coinfection with EBV and HPV may increase the risk of UCC development. The hypothesis is that the two viruses could act synergistically, with EBV potentially promoting HPV-induced cervical carcinogenesis (Table 2; da Carvalho and de Melo, 2019; Blanco et al., 2020). Co-infection with EBV, HPV, and human immunodeficiency virus (HIV) could potentially increase the risk of UCC development (Denny et al., 2008; Kelly et al., 2018). Infection with these high-risk types can lead to the formation of abnormal cervical cells, which may progress to UCC if not detected and treated early. HIV infection impairs the immune system, making it more difficult for the body to fight off infections, including HPV. Women with HIV are more likely to have persistent HPV infections, which can increase the risk of developing UCC (Clifford et al., 2017). Additionally, HIV-infected women are more likely to have faster progression from pre-cancerous lesions to invasive UCC compared to women without HIV. Although EBV is not directly linked to UCC, co-infection with HIV could further increase the risk due to the impaired immune response. Co-infections can complicate the clinical picture, and it is essential for healthcare providers (Table 2; Feng M. et al., 2021). HPV, EBV, and KSHV coinfections can stimulate angiogenesis (Figure 1), the formation of new blood vessels, by upregulating pro-angiogenic factors (e.g., VEGF and IL-8), which may promote tumor growth and metastasis (Dai et al., 2018; Hemmat and Bannazadeh Baghi, 2019). Upon coinfection, EBV and HPV oncoproteins have been shown to cooperatively activate several critical signaling pathways, including PI3K/AKT, MAPK/ERK, JAK/STAT, β-catenin, and p53 (Vranic et al., 2018). These pathways are known to regulate various cellular processes, such as cell survival, proliferation, differentiation, and migration, which are often dysregulated in cancer. Consequently, the simultaneous activation of these pathways by EBV and HPV oncoproteins can contribute to enhanced UCC development and progression.

Given the current state of research, the direct relationship between EBV and UCC risk remains unclear. Further studies are needed to investigate the potential association between EBV and uterine cancer, as well as the underlying mechanisms involved if such a link exists.

## HBV and HCV infection increases the development of UCC risk indirectly

Hepatitis B viruses is a DNA virus that primarily infects the liver and can cause both acute and chronic hepatitis. HBV is a well-established risk factor for liver cancer, specifically hepatocellular carcinoma (HCC). However, the association between HBV infection and uterine cancer, particularly UCC, is not well-established. There is limited research on the potential relationship between HBV and uterine cancer, and the available studies do not provide sufficient evidence to establish a clear link between HBV infection and the risk of developing uterine cancer (Table 3). It is important to note that HBsAg (hepatitis B surface antigen) is associated with hepatitis B infection, which primarily affects the liver, while HPV is the main causative agent for UCC. Both HBV and HPV infection can potentially contribute to a weakened immune system, which may make it more difficult for the body to fight off other infections, including HPV. A weakened immune system could theoretically increase the risk of developing UCC in HPV positive individuals, but the primary risk factor remains the HPV infection itself (Table 3; Ferber et al., 2003; Luo et al., 2022). Although there is not a direct causal link between HBV infection and squamous cell UCC, some studies have suggested a potential association between the two. Serological markers of HBV infection, such as HBsAg, hepatitis B e antigen (HBeAg), and hepatitis B core antibody (anti-HBc), can provide information about the presence and stage of HBV infection in an individual. These markers may have prognostic value in certain cancer types (Table 3; Feng X. et al., 2021). The presence of HBsAg in serum indicates an ongoing infection, either acute or chronic (Lok and McMahon, 2009). HBeAg is a secreted viral protein that reflects active viral replication and is associated with high infectivity (Hadziyannis, 1995). Anti-HBc is an antibody produced in response to the HBcAg, which is a component of the viral nucleocapsid. The presence of anti-HBc indicates previous exposure to HBV, either resolved or ongoing (Chu and Liaw, 2010). In a study involving 277 cervical cancer patients, the seropositivity rates for HBsAg, HBeAg, and anti-HBc were found to be 4.33, 0.72, and 13.00%, respectively, indicating a potential relationship between HBV infection and cervical cancer (Wu et al., 2021). The detection of HBsAg and HBcAg in a subset of cervical cancer cases with seropositive HBsAg, as well as the increased risk of cervical cancer in individuals with both HBsAg and HPV positive, underscores the potential interplay between HBV infection and HPV in cervical cancer development. This observation highlights the importance of considering co-infections when examining UCC risk factors (Luo et al., 2022).

**TABLE 3:** The indirect association between hepatitis B virus (HBV) and hepatitis C virus (HCV) infections and the increased risk of developing uterine cervical Cancer (UCC).

Country and date	Virus strains	Main findings	Applications	References
Luo et al. (2022)–China	HBC and HCV	HBsAg and HPV positive had an increased risk of UCC	HBV infection should be avoided.	Luo et al., 2022
Feng X. et al. (2021)–China	HBV	HBV infection is associated with poor prognosis in patients with primary UCC.	The main application is investigating the prognostic impact of hepatitis B virus (HBV) infection in patients with primary UCC.	Feng X. et al., 2021
Chhetri (2010)–India	HCV	A case report suggests an association between HCV infection and carcinoma cervix, highlighting the need for further studies.	This article focuses on a case report and brief review of the literature on the association between chronic hepatitis C virus (HCV) infection and carcinoma cervix.	Chhetri, 2010
Ferber et al. (2003)–United States	HBV	Integrations of HBV and HPV into the human telomerase reverse transcriptase (hTERT) gene were found in liver and UCC, suggesting a possible oncogenic mechanism.	The study examines the integrations of HBV and human papillomavirus (HPV) into the human telomerase reverse transcriptase (hTERT) gene in liver and UCC.	Ferber et al., 2003
Mahmood et al. (2002)–United Kingdom	HCV	HCV RNA was detected in normal cervical smears of HCV-seropositive patients, indicating a possible role for HCV in cervical pathogenesis.	This research aims to detect HCV RNA in normal cervical smears of HCV-seropositive patients.	Mahmood et al., 2002
Shiels et al. (2013)–United States	None mentioned	The non-Hodgkin lymphoma epidemic in the United States was analyzed, with results suggesting factors other than HIV playing a significant role in its development.	The article analyzes the epidemic of non-Hodgkin lymphoma in the United States, disentangling the effect of HIV from 1992 to 2009.	Shiels et al., 2013
Engels et al. (2010)–Republic of Korea	HBV	HBV infection was found to be associated with an increased risk of non-Hodgkin lymphoma in a South Korean cohort study.	This cohort study investigates the association between HBV infection and risk of non-Hodgkin lymphoma in Republic of Korea.	Engels et al., 2010
Kumar et al. (2018)–India	HBV	Multiple idiopathic cervical root resorptions were reported in patients with HBV infection, suggesting a potential association.	The research examines the occurrence of multiple idiopathic cervical root resorptions in patients with HBV infection.	Kumar et al., 2018
Heffernan et al. (2010)–Australia	None mentioned	The global reduction of UCC through HPV vaccines is discussed, with insights drawn from HBV vaccine experience.	The main application is exploring global reduction of UCC with HPV vaccines, drawing insights from the hepatitis B virus vaccine experience.	Heffernan et al., 2010
Wu et al. (2021)–China	HBV	Serological markers of HBV infection have prognostic value in squamous cell UCC.	The study investigates the prognostic value of serological markers of HBV infection in squamous cell UCC.	Wu et al., 2021
Siu et al. (2007)–Hong Kong SAR, China	HBV	Patients with malignant or pre-malignant cervical lesions were found to have an increased risk of becoming hepatitis B carriers.	This research explores the increased risk of becoming an HBV carrier in patients with malignant or pre-malignant cervical lesions.	Siu et al., 2007
Li et al. (2015)–China	HBV	Up-regulation of human UCC proto-oncogene contributes to HBV-induced malignant transformation of hepatocytes by down-regulating E-cadherin.	The article examines the role of human UCC proto-oncogene in contributing to HBV-induced malignant transformation of hepatocytes by down-regulating E-cadherin.	Li et al., 2015
Leroux-Roels et al. (2011)–Belgium	HBV	The immunogenicity and safety of hepatitis B and human papillomavirus type 16/18 AS04-adjuvanted UCC vaccines were found to be satisfactory when coadministered in an accelerated schedule.	The study evaluates the immunogenicity and safety of the hepatitis B vaccine given in an accelerated schedule coadministered with the HPV type 16/18 AS04-adjuvanted UCC vaccine.	Leroux-Roels et al., 2011
Dimond et al. (2021)–United States	HBV	A fatal hepatitis B reactivation case was reported in a patient receiving chemoradiation for UCC, highlighting the need for careful monitoring and management of HBV during treatment.	This case report discusses fatal hepatitis B reactivation in a patient receiving chemoradiation for UCC.	Dimond et al., 2021

Hepatitis C viruses is a well-known risk factor for liver cancer, particularly hepatocellular carcinoma. However, its association with uterine cancer, particularly UCC, is unclear and not well-established (Table 3; Mahmood et al., 2002; Chhetri, 2010). The prevalence of HBV and HCV infections was found among UCC patients compared to the general population. The association between chronic HBV infection and cervical cancer disappears among HPV-positive patients but remains significant for patients younger than 50 years after adjusting for HPV infection and parity. Co-infection with sexually transmitted infections (STIs), such as HIV, HBV, HCV, can also increase the risk of developing UCC. The seroprevalence of STIs among cervical cancer suspected women in Ethiopia, highlights a significant public health concern. The authors report an overall STI prevalence of 16.6% (67/403) in the study population, with the prevalence of HIV, HBV, HCV, and syphilis being 8.9, 2.5, 1, and 7.2%, respectively. This information helps to bridge the existing knowledge gap concerning the burden of coinfection in this UCC population (Abebe et al., 2021).

The clinical importance of EBV, HBV, and HCV in the etiology of cervical cancer has become an area of increasing interest among researchers and clinicians. The exact mechanisms by which EBV may contribute to cervical cancer development remain unclear, but possible explanations include the induction of genomic instability, inhibition of apoptosis, and promotion of cell proliferation (Figure 1). The possible mechanisms through which HBV may contribute to chronic inflammation, immune suppression, or molecular mimicry. Further investigation is warranted to determine the clinical relevance of HBV in the context of cervical cancer and to assess whether prevention and control of HBV infection may have implications for cervical cancer risk reduction. Similar to HBV, HCV is primarily implicated in liver diseases but has also been suggested as a possible co-factor in cervical cancer development. Some studies have reported an increased prevalence of HCV infection among cervical cancer patients, raising the possibility of an association between the two conditions. The potential mechanisms linking HCV to cervical cancer remain speculative and may involve chronic inflammation or immune dysregulation. In conclusion, the clinical importance of EBV, HBV, and HCV in cervical cancer etiology remains an emerging area of investigation. Although these viruses are not considered primary causative agents of cervical cancer, their potential role in the development of this malignancy warrants further study. Understanding the mechanisms through which these viruses may contribute to cervical carcinogenesis could have important implications for the prevention, diagnosis, and treatment of cervical cancer, ultimately improving patient outcomes and public health strategies.

## Other virus strains and UCC risk

Human cytomegalovirus (HCMV) has been associated with various malignancies, including glioblastoma and prostate cancer. It has been detected in some UCC tissues (Kienka et al., 2019; Khashman et al., 2020b; Ghadicolaee et al., 2021), but the relationship is not well-established. Human T-cell lymphotropic virus type 1 (HTLV-1) is a retrovirus known to be associated with adult T-cell leukemia/lymphoma (ATL) and a neurological disorder called HTLV-1-associated myelopathy/tropical spastic paraparesis (HAM/TSP). Although HTLV-1 has not been directly implicated in UCC, some studies have suggested a potential link between HTLV-1 infection and an increased risk of developing UCC (Schierhout et al., 2020). The connection between HTLV-1 and UCC is not as well-established as that of HPV, which is the primary cause of UCC. However, there is some evidence to suggest that HTLV-1 infection might influence the persistence or progression of HPV infection, potentially increasing the risk of UCC in HTLV-1-infected women (Ibrahim Jaber and Qasim Dhumad, 2022; Rosadas and Taylor, 2022). Additionally, it has been suggested that HTLV-1 might indirectly contribute to UCC development by impairing the immune system and reducing the body’s ability to control HPV infection (Ibrahim Jaber and Qasim Dhumad, 2022).

Kaposi’s sarcoma-associated herpesvirus (KSHV), also known as human herpesvirus 8 (HHV-8), is a virus primarily associated with Kaposi’s sarcoma, primary effusion lymphoma, and multicentric Castleman’s disease. While KSHV has been extensively studied in relation to these conditions, its direct association with UCC is not well-established. Although KSHV and HPV are both viruses that can cause cancer, their roles in the development of UCC are distinct. HPV is considered the primary etiological agent for UCC, while KSHV has not been definitively linked to this malignancy. There have been some studies investigating the potential role of KSHV in UCC (Mukerebe, 2022), but the evidence is not yet strong enough to establish a direct association. Further research is needed to determine if KSHV plays a role in the development or progression of UCC or if it has any interaction with HPV in cervical carcinogenesis.

Human herpesvirus (HHV), particularly HHV-2, also known as herpes simplex virus type 2 (HSV-2), has been suggested to play a role in the development of UCC (Mukerebe, 2022). HSV-2 is primarily responsible for genital herpes infections, which can cause genital ulcers and increase the risk of acquiring other sexually transmitted infections (STIs), including HPV (Moharreri and Sohrabi, 2021). While HSV-2 itself is not considered a direct cause of UCC, its association with genital ulcers and the increased risk of acquiring HPV could potentially contribute to the development of UCC. Furthermore, HSV-2 infection might cause local inflammation and immunosuppression in the cervical area, which could facilitate the persistence and progression of HPV infection, ultimately leading to UCC (Vitali et al., 2020).

Human immunodeficiency virus is a virus that attacks the immune system, specifically the CD4 + T cells, which play a crucial role in immune response. HIV infection can progress to acquired immunodeficiency syndrome (AIDS) when the immune system is severely damaged, leaving the individual vulnerable to various opportunistic infections and cancers. There is a strong association between HIV and UCC. HIV infection weakens the immune system, making it difficult for the body to fight off HPV infections and prevent the progression of pre-cancerous lesions to UCC (Marima et al., 2021; Stelzle et al., 2021). HIV-infected women have a higher prevalence of HPV infection and are more likely to be infected with multiple high-risk HPV types. They are also more likely to have persistent HPV infections, which increases the risk of developing UCC (D’andrea et al., 2019). HIV-infected women tend to have a faster progression from HPV infection to the development of precancerous lesions and invasive UCC compared to women without HIV infection (Moscicki et al., 2019; Tawe et al., 2020). HIV-infected women may have a poorer response to UCC treatments, such as surgery, radiation, and chemotherapy, due to their compromised immune systems. To reduce the risk of UCC in HIV-infected women, regular UCC screening (e.g., Pap smears or HPV tests) is recommended. Additionally, the administration of the HPV vaccine can help protect against the high-risk HPV types responsible for UCC. Antiretroviral therapy (ART) can also help to improve the immune system in HIV-infected individuals, potentially reducing the risk of developing UCC (Shin et al., 2019). It is important to note that the link between many types of viruses and UCC is still not fully understood, and more research is needed to establish a clear relationship between the two. HPV remains the primary risk factor for UCC, and the prevention and control of HPV infection through vaccination and screening are the most effective strategies for reducing the incidence of UCC. More research is needed to determine the exact relationship between different virus types and UCC, as the current evidence is limited and inconclusive.

## The future challenge for the viral etiology of UCC

The future challenges for the viral etiology of UCC, particularly in relation to HPV, include (1) Improving vaccination coverage, although the HPV vaccine has proven effective in reducing the prevalence of high-risk HPV strains associated with UCC, improving vaccination coverage globally remains a challenge, especially in low- and middle-income countries where the burden of UCC is high; (2) Understanding HPV and other viral coinfections, such as EBV, may potentially play a role in UCC development. Further research is needed to elucidate the mechanisms and significance of these coinfections; (3) expanding screening programs: screening programs using HPV DNA testing have shown promise in detecting precancerous lesions, but implementing and expanding these programs worldwide, particularly in resource-limited settings, remains a challenge; (4) addressing disparities in access to care, disparities in access to preventive care, such as vaccination and screening, contribute to the high burden of UCC in certain populations. Efforts should be made to address socioeconomic, cultural, and logistical barriers to care; (5) developing novel therapeutic approaches, although current treatments for UCC, such as surgery, radiation, and chemotherapy, can be effective, there is a need for novel therapies that target the viral etiology of the disease, such as antiviral drugs and immunotherapies; (6) enhancing public awareness and education, public awareness and understanding of the role of HPV in UCC and the importance of vaccination and screening are essential to reduce the burden of the disease. Educational campaigns targeting various populations can help increase vaccine uptake and participation in screening programs; (7) investigating the role of viral genetic variation, the role of genetic variation within HPV types and its impact on UCC risk and vaccine efficacy needs further investigation; (8) Studying the impact of the HPV vaccine on non-cervical HPV-associated cancers, the HPV vaccine has the potential to prevent other HPV-associated cancers, such as oropharyngeal, anal, and penile cancers. Further research is needed to understand the long-term impact of vaccination on these cancers; (9) Addressing vaccine hesitancy: Vaccine hesitancy remains a challenge in certain populations, and efforts should be made to address misconceptions and promote confidence in the HPV vaccine, and (10) Long-term monitoring and surveillance, continued monitoring and surveillance of HPV prevalence, vaccine efficacy, and UCC incidence are crucial to assess the long-term impact of vaccination and screening programs and inform public health policy.

## Author contributions

DC and TL: review conception, design, data collection, and quality analysis. TL and YY: the data extraction of the included studies, analysis, and interpretation of results. DC and YY: draft manuscript preparation and the critical revision of the manuscript. All authors reviewed the results and approved the final manuscript.

## References

[B1] Abebe M. Eshetie S. Tessema B. (2021). Prevalence of sexually transmitted infections among cervical cancer suspected women at University of Gondar Comprehensive Specialized Hospital, North-west Ethiopia. *BMC Infect. Dis.* 21:378. 10.1186/s12879-021-06074-y 33888090 PMC8063310

[B2] Abudoukadeer A. Niyazi M. Aikula A. Kamilijian M. Sulaiman X. Mutailipu A. (2015). Association of EBV and HPV co-infection with the development of cervical cancer in ethnic Uyghur women. *Eur. J. Gynaecol. Oncol.* 36 546–550. 26513880

[B3] Abuelo C. Levinson K. Salmeron J. Sologuren C. Fernandez M. Belinson J. (2014). The Peru cervical cancer screening study (PERCAPS): The design and implementation of a mother/daughter screen, treat, and vaccinate program in the Peruvian jungle. *J. Commun. Health* 39 409–415. 10.1007/s10900-013-9786-6 24276617 PMC4543313

[B4] Ackermann S. Renner S. Fasching P. Poehls U. Bender H. Beckmann M. (2005). Awareness of general and personal risk factors for uterine cancer among healthy women. *Eur. J. Cancer Prev.* 14 519–524. 16284496 10.1097/00008469-200512000-00005

[B5] Arbyn M. Castle P. Schiffman M. Wentzensen N. Heckman-Stoddard B. Sahasrabuddhe V. (2022a). Meta-analysis of agreement/concordance statistics in studies comparing self-vs clinician-collected samples for HPV testing in cervical cancer screening. *Int. J. Cancer* 151 308–312. 35179777 10.1002/ijc.33967

[B6] Arbyn M. Simon M. de Sanjosé S. Clarke M. Poljak P. Rezhake R. (2022b). Accuracy and effectiveness of HPV mRNA testing in cervical cancer screening: A systematic review and meta-analysis. *Lancet Oncol.* 23 950–960. 35709810 10.1016/S1470-2045(22)00294-7

[B7] Arbyn M. Smith S. Temin S. Sultana F. Castle P. (2018). Detecting cervical precancer and reaching underscreened women by using HPV testing on self samples: Updated meta-analyses. *BMJ* 363:k4823. 10.1136/bmj.k4823 30518635 PMC6278587

[B8] Arbyn M. Verdoodt F. Snijders P. Verhoef V. Suonio E. Dillner L. (2014). Accuracy of human papillomavirus testing on self-collected versus clinician-collected samples: A meta-analysis. *Lancet Oncol.* 15 172–183. 24433684 10.1016/S1470-2045(13)70570-9

[B9] Arrossi S. Ramos S. Straw C. Thouyaret L. Orellana L. (2016). HPV testing: A mixed-method approach to understand why women prefer self-collection in a middle-income country. *BMC Public Health* 16:832. 10.1186/s12889-016-3474-2 27538390 PMC4990977

[B10] Awasthi P. Dwivedi M. Kumar D. Hasan S. (2023). Insights into intricacies of the latent membrane protein-1 (LMP-1) in EBV-associated cancers. *Life Sci.* 313:121261. 10.1016/j.lfs.2022.121261 36493876

[B11] Basoya S. Anjankar A. (2022). Cervical cancer: Early detection and prevention in reproductive age group. *Cureus* 14:e31312. 10.7759/cureus.31312 36514565 PMC9735321

[B12] Bennett K. Waller J. Chorley A. Ferrer R. Haddrell J. Marlow L. (2018). Barriers to cervical screening and interest in self-sampling among women who actively decline screening. *J. Med. Screen.* 25 211–217. 10.1177/0969141318767471 29649936 PMC6262593

[B13] Bhatla N. Singhal S. (2020). Primary HPV screening for cervical cancer. *Best Pract. Res. Clin. Obstet. Gynaecol.* 65 98–108. 32291178 10.1016/j.bpobgyn.2020.02.008

[B14] Blanco R. Carrillo-Beltrán D. Osorio J. Calaf G. Aguayo F. (2020). Role of Epstein-Barr virus and human papillomavirus coinfection in cervical cancer: Epidemiology, mechanisms and perspectives. *Pathogens* 9:685. 10.3390/pathogens9090685 32839399 PMC7557835

[B15] Bohn J. Hernandez-Zepeda M. Hersh A. Munro E. Kahn J. Caughey A. (2022). Does obesity influence the preferred treatment approach for early-stage cervical cancer? A cost-effectiveness analysis. *Int. J. Gynecol. Cancer* 32 133–140. 10.1136/ijgc-2021-003004 34887286

[B16] Bosch F. Lorincz A. Muñoz N. Meijer C. Shah K. (2002). The causal relation between human papillomavirus and cervical cancer. *J. Clin. Pathol.* 55 244–265. 11919208 10.1136/jcp.55.4.244 PMC1769629

[B17] Brewster W. Monk B. Burger R. Bergen S. Wilczynski S. (1999). Does human papillomavirus have a role in cancers of the uterine corpus? *Gynecol. Oncol.* 75 51–54. 10502425 10.1006/gyno.1999.5534

[B18] Brown D. Kjaer S. Sigurdsson K. Iversen O. Hernandez-Avila M. Wheeler C. (2009). The impact of quadrivalent human papillomavirus (HPV; types 6, 11, 16, and 18) L1 virus-like particle vaccine on infection and disease due to oncogenic nonvaccine HPV types in generally HPV-naive women aged 16–26 years. *J. Infect. Dis.* 199 926–935. 10.1086/597307 19236279

[B19] Cameron J. Dennis D. Herrel N. Chapple A. Hagensee M. (2020). Risk of abnormal cervical cytology in HIV-infected women testing positive for both human papillomavirus and Epstein-Barr virus in genital tract specimens. *Cancer Causes Control* 31 365–375. 10.1007/s10552-020-01287-z 32112173 PMC8432267

[B20] Castellsagué X. Menéndez C. Loscertales M. P. Kornegay J. R. dos Santos F. Gómez-Olivé F. X. (2001). Human papillomavirus genotypes in rural Mozambique. *Lancet* 358, 1429–1430. 10.1016/S0140-6736(01)06523-0 11705494

[B21] Castro D. Vera J. Soto-Becerra P. López-Ilasaca M. Yabar A. Cámara A. (2020). Epstein-Barr virus and its prognostic value in a cohort of Peruvian women with cervical cancer. *medRxiv [Preprint]* 10.1101/2020.08.04.20167841

[B22] Chandra S. Sarkar S. Mandal P. (2022). Identification of novel genetic and epigenetic regulators of different tissue types of cervical cancer. *J. Obstet. Gynaecol. Res.* 48 3179–3190. 10.1111/jog.15454 36184073

[B23] Chen W. Sun K. Zheng R. Zeng H. Zhang S. Xia C. (2018). Cancer incidence and mortality in China, 2014. *Chin. J. Cancer Res.* 30 1–12. 29545714 10.21147/j.issn.1000-9604.2018.01.01 PMC5842223

[B24] Chhetri M. (2010). Chronic hepatitis C virus infection and carcinoma cervix–report of a case and brief review of literature. *Apollo Med.* 7 61–63.

[B25] Choi S. Ismail A. Pappas-Gogos G. Boussios S. (2023). HPV and cervical cancer: A review of epidemiology and screening uptake in the UK. *Pathogens* 12:298. 10.3390/pathogens12020298 PMC9960303 36839570

[B26] Chu C. Liaw Y. (2010). Hepatitis B surface antigen seroclearance during chronic HBV infection. *Antivir. Ther.* 15 133–143. 20386068 10.3851/IMP1497

[B27] Clifford G. Tully S. Franceschi S. (2017). Carcinogenicity of human papillomavirus (HPV) types in HIV-positive women: A meta-analysis from HPV infection to cervical cancer. *Clin. Infect. Dis.* 64 1228–1235. 10.1093/cid/cix135 28199532 PMC5399941

[B28] Coffey K. Gaitskell K. Beral V. Canfell K. Green J. Reeves G. (2016). Past cervical intraepithelial neoplasia grade 3, obesity, and earlier menopause are associated with an increased risk of vulval cancer in postmenopausal women. *Br. J. Cancer* 115 599–606. 10.1038/bjc.2016.165 27336599 PMC4997536

[B29] Costa S. Verberckmoes B. Castle P. Arbyn M. (2023). Offering HPV self-sampling kits: An updated meta-analysis of the effectiveness of strategies to increase participation in cervical cancer screening. *Br. J. Cancer* 128 805–813. 10.1038/s41416-022-02094-w 36517552 PMC9977737

[B30] Cuzick J. Clavel C. Petry K. Meijer C. Hoyer H. Ratnam S. (2006). Overview of the European and North American studies on HPV testing in primary cervical cancer screening. *Int. J. Cancer* 119 1095–1101. 10.1002/ijc.21955 16586444

[B31] da Carvalho M. de Melo Y. (2019). Association between human papillomavirus and Epstein-Barr virus infections and cancer of the uterine cervix. *Crit. Rev. Oncog.* 24 379–383. 32421992 10.1615/CritRevOncog.2019031545

[B32] Dai L. Zhao M. Jiang W. Lin Z. Del Valle L. Qin Z. (2018). KSHV co-infection, a new co-factor for HPV-related cervical carcinogenesis? *Am. J. Cancer Res.* 8 2176–2184. 30555737 PMC6291645

[B33] D’andrea F. Pellicanò G. Venanzi Rullo E. D’ALEO F. Facciolà A. Mical C. (2019). Cervical cancer in women living with HIV: A review of the literature. *World Cancer Res. J.* 6:e1224.

[B34] Dau H. Trawin J. Nakisige C. Payne B. Vidler M. Singer J. (2023). The social and economic impacts of cervical cancer on women and children in low- and middle-income countries: A systematic review. *Int. J. Gynaecol. Obstet.* 160 751–761. 10.1002/ijgo.14395 35962711

[B35] de Lima M. Neto P. Lima L. Gonçalves Júnior J. Teixeira Junior A. Teodoro I. (2018). Association between Epstein-Barr virus (EBV) and cervical carcinoma: A meta-analysis. *Gynecol. Oncol.* 148 317–328. 29021084 10.1016/j.ygyno.2017.10.005

[B36] De Oliveira D. Monteiro T. De Melo W. Moreira M. Alvarenga M. Bacchi C. (1999). Lack of Epstein-Barr virus infection in cervical carcinomas. *Arch. Pathol. Lab. Med.* 123 1098–1100. 10539915 10.5858/1999-123-1098-LOEBVI

[B37] de Sanjose S. Quint W. Alemany L. Geraets D. Klaustermeier J. Lloveras B. (2010). Human papillomavirus genotype attribution in invasive cervical cancer: A retrospective cross-sectional worldwide study. *Lancet Oncol.* 11 1048–1056. 20952254 10.1016/S1470-2045(10)70230-8

[B38] Denny L. Boa R. Williamson A. Allan B. Hardie D. Stan R. (2008). Human papillomavirus infection and cervical disease in human immunodeficiency virus-1-infected women. *Obstet. Gynecol.* 111 1380–1387. 10.1097/AOG.0b013e3181743327 18515522

[B39] Dimond C. Negroiu A. Hughes D. Patel J. (2021). Fatal hepatitis B reactivation in a patient receiving chemoradiation for cervical cancer. *J. Oncol. Pharm. Pract.* 27 1296–1301. 10.1177/1078155220964256 33054690

[B40] Drolet M. Bénard É Pérez N. Brisson M. Hpv Vaccination Impact Study Group. (2019). Population-level impact and herd effects following the introduction of human papillomavirus vaccination programmes: Updated systematic review and meta-analysis. *Lancet* 394 497–509. 10.1016/S0140-6736(19)30298-3 31255301 PMC7316527

[B41] Elfström K. Smelov V. Johansson A. Eklund C. Nauclér P. Arnheim-Dahlström L. (2014). Long term duration of protective effect for HPV negative women: Follow-up of primary HPV screening randomised controlled trial. *BMJ* 348:g130. 10.1136/bmj.g130 24435414 PMC3898575

[B42] Engels E. Cho E. Jee S. (2010). Hepatitis B virus infection and risk of non-Hodgkin lymphoma in South Korea: A cohort study. *Lancet Oncol.* 11 827–834. 20688564 10.1016/S1470-2045(10)70167-4 PMC2933963

[B43] Evans A. Salnikov M. Gameiro S. Maleki Vareki S. Mymryk J. (2022). HPV-positive and-negative cervical cancers are immunologically distinct. *J. Clin. Med.* 11:4825. 10.3390/jcm11164825 36013065 PMC9410291

[B44] Feng M. Duan R. Gao Y. Zhang H. Qiao Y. Li Q. (2021). Role of Epstein-Barr virus and human papillomavirus coinfection in cervical intraepithelial neoplasia in Chinese women living with HIV. *Front. Cell. Infect. Microbiol.* 11:703259. 10.3389/fcimb.2021.703259 34557425 PMC8453025

[B45] Feng X. Lu H. Wei Y. Guan M. Wang J. Liu C. (2021). Prognostic impact of hepatitis B virus infection in patients with primary cervical cancer. *Cancer Med.* 10 8310–8319. 10.1002/cam4.4358 34672431 PMC8633261

[B46] Ferber M. J. Montoya D. P. Yu C. Aderca I. McGee A. Thorland E. C. (2003). Integrations of the hepatitis B virus (HBV) and human papillomavirus (HPV) into the human telomerase reverse transcriptase (hTERT) gene in liver and cervical cancers. *Oncogene* 22 3813–3820. 10.1038/sj.onc.1206528 12802289

[B47] Ferrall L. Lin K. Roden R. Hung C. Wu T. (2021). Cervical cancer immunotherapy: Facts and hopes. *Clin. Cancer Res.* 27 4953–4973. 10.1158/1078-0432.CCR-20-2833 33888488 PMC8448896

[B48] Figueiredo D. Ribeiro I. Penedones A. Mendes D. Alves C. Batel-Marques F. (2023). Performance of Aptima-HPV in the cervical cancer screening program of Portugal: A cost-analysis. *BMC Womens Health* 23:96. 10.1186/s12905-023-02219-0 36894908 PMC9999620

[B49] Fine P. Eames K. Heymann D. (2011). “Herd immunity”: A rough guide. *Clin. Infect. Dis.* 52 911–916. 10.1093/cid/cir007 21427399

[B50] Frumovitz M. Jhingran A. Soliman P. Klopp A. Schmeler K. Eifel P. (2014). Morbid obesity as an independent risk factor for disease-specific mortality in women with cervical cancer. *Obstet. Gynecol.* 124 1098–1104. 10.1097/AOG.0000000000000558 25415160 PMC4249728

[B51] Fujii T. Okabe A. Kaneda A. (2022). Epigenetic contribution to tumorigenesis of host cells by Epstein-Barr virus infection. *Chiba Med. J.* 98 1–7.

[B52] Ghadicolaee S. Pazhoohan M. Hasanzadeh A. Nematolahi M. Yahyapour Y. Ranaee M. (2021). Low frequency of human cytomegalovirus in cancerous and precancerous cervical samples of Iranian women. *Future Virol.* 16 399–405.

[B53] Gilham C. Sargent A. Crosbie E. Peto J. (2023). Long-term risks of invasive cervical cancer following HPV infection: Follow-up of two screening cohorts in Manchester. *Br. J. Cancer* 128 1933–1940. 10.1038/s41416-023-02227-9 36959379 PMC10147679

[B54] Giuliano A. Sedjo R. Roe D. Harri R. Baldwi S. Papenfuss M. (2002). Clearance of oncogenic human papillomavirus (HPV) infection: Effect of smoking (United States). *Cancer Causes Control* 13 839–846. 12462549 10.1023/a:1020668232219

[B55] Gupta S. Palmer C. Bik E. Cardenas J. Nuñez H. Kraal L. (2018). Self-sampling for human papillomavirus testing: Increased cervical cancer screening participation and incorporation in international screening programs. *Front. Public Health* 6:77. 10.3389/fpubh.2018.00077 29686981 PMC5900042

[B56] Hadziyannis S. (1995). Hepatitis B e antigen negative chronic hepatitis B: From clinical recognition to pathogenesis and treatment. *Viral Hepat Rev.* 1 7–36.

[B57] Harper D. DeMars L. (2017). HPV vaccines–a review of the first decade. *Gynecol. Oncol.* 146 196–204. 28442134 10.1016/j.ygyno.2017.04.004

[B58] Heffernan M. Garland S. Kane M. (2010). Global reduction of cervical cancer with human papillomavirus vaccines: Insights from the hepatitis B virus vaccine experience. *Sex. Health* 7 383–390. 10.1071/SH09134 20719231

[B59] Hemmat N. Bannazadeh Baghi H. (2019). Association of human papillomavirus infection and inflammation in cervical cancer. *Pathog. Dis.* 77:ftz048. 10.1093/femspd/ftz048 31504464

[B60] Hildesheim A. Herrero R. Castle P. E. Wacholder S. Bratti M. C. Sherman M. E. (2001). HPV co-factors related to the development of cervical cancer: Results from a population-based study in Costa Rica. *Br. J. Cancer* 84 1219–1226. 10.1054/bjoc.2001.1779 11336474 PMC2363883

[B61] Hoseini Tabatabaie F. Hosseini S. Hashemi S. Safaie A. Sarvari J. (2023). A preliminary sequence analysis of the Epstein-Barr virus nuclear antigen 1 (EBNA1) carboxy-terminal region in cervical and ovarian cancers. *Iran. J. Pathol.* 18, 24–32. 10.30699/ijp.2023.551761.2872 PMC10293606 37383155

[B62] Huh W. Joura E. Giuliano A. Iversen O. de Andrade R. Ault K. (2017). Final efficacy, immunogenicity, and safety analyses of a nine-valent human papillomavirus vaccine in women aged 16–26 years: A randomised, double-blind trial. *Lancet* 390 2143–2159. 10.1016/S0140-6736(17)31821-4 28886907

[B63] Ibrahim Jaber S. Qasim Dhumad B. (2022). Expression of HPV-16 L1 gene human papillomavirus (HTLV-1) identified by pap smear. *Arch. Razi Inst.* 77 2125–2130. 37274879 10.22092/ARI.2022.359292.2397 PMC10237539

[B64] Insinga R. Dasbach E. Myers E. (2003). The health and economic burden of genital warts in a set of private health plans in the United States. *Clin. Infect. Dis.* 36 1397–1403. 10.1086/375074 12766834

[B65] Iversen L. Fielding S. Lidegaard O. Hannaford P. (2021). Contemporary hormonal contraception and cervical cancer in women of reproductive age. *Int. J. Cancer* 10.1002/ijc.33585 [Epub ahead of print]. 33818778

[B66] Joura E. Giuliano A. Iversen O. Bouchard C. Mao C. Mehlsen J. (2015). A 9-valent HPV vaccine against infection and intraepithelial neoplasia in women. *N. Engl. J. Med.* 372 711–723. 25693011 10.1056/NEJMoa1405044

[B67] Kamal M. Lameiras S. Deloger M. Morel A. Vacher S. Lecerf C. (2021). Human papilloma virus (HPV) integration signature in cervical cancer: Identification of MACROD2 gene as HPV hot spot integration site. *Br. J. Cancer* 124 777–785. 33191407 10.1038/s41416-020-01153-4 PMC7884736

[B68] Kelly H. Weiss H. Benavente Y. de Sanjose S. Mayaud P. (2018). Association of antiretroviral therapy with high-risk human papillomavirus, cervical intraepithelial neoplasia, and invasive cervical cancer in women living with HIV: A systematic review and meta-analysis. *Lancet HIV* 5 e45–e58. 10.1016/s2352-3018(17)30149-2 29107561 PMC5757426

[B69] Khashman B. Al-Zahawi F. Alwandawi T. Ali M. J. (2020a). Expression of EBV latent membrane protein 1 (LMP1) in Iraqi women with cervical carcinoma. *Biochem. Cell. Arch.* 20:3263.

[B70] Khashman B. Karim S. Alhilli H. Ali M. (2020b). Possible role of HCMV infection on the development of HPV positive cervical carcinoma in a group of Iraqi women. *Biochem. Cell. Arch.* 20 1549–1552.

[B71] Kienka T. Varga M. Caves J. Smith J. Sivaraman V. (2019). Epstein-Barr virus, but not human cytomegalovirus, is associated with a high-grade human papillomavirus–associated cervical lesions among women in North Carolina. *J. Med. Virol.* 91 450–456. 30307626 10.1002/jmv.25336 PMC6331249

[B72] Kim J. Kim B. Jeon D. Lee C. Roh J. Kim J. (2020). Type-specific viral load and physical state of HPV type 16, 18, and 58 as diagnostic biomarkers for high-grade squamous intraepithelial lesions or cervical Cancer. *Cancer Res. Treat.* 52 396–405. 31476849 10.4143/crt.2019.152 PMC7176961

[B73] Kolesnik M. Stepien E. Polz-Dacewicz M. (2021). The role of microRNA (miRNA) as a biomarker in HPV and EBV-related cancers. *J. Preclin. Clin. Res.* 15 104–110.

[B74] Korsakov A. Kryukova A. Troshin V. Milushkina O. Lagerev D. (2022). Cervical and endometrial cancer incidence in the female population from the bryansk region living in conditions of chemical, radioactive and combined environmental contamination (2000-2020). *Life* 12:1488. 10.3390/life12101488 36294923 PMC9605682

[B75] Kreimer A. Struyf F. Del Rosario-Raymundo M. Hildesheim A. Skinner S. Wacholder S. (2015). Efficacy of fewer than three doses of an HPV-16/18 AS04-adjuvanted vaccine: Combined analysis of data from the Costa Rica vaccine and PATRICIA trials. *Lancet Oncol.* 16 775–786. 10.1016/S1470-2045(15)00047-9 26071347 PMC4498478

[B76] Kumar V. Chawla A. Kaur A. (2018). Multiple idiopathic cervical root resorptions in patients with hepatitis B virus infection. *J. Endod.* 44 1575–1577. 10.1016/j.joen.2018.06.017 30144987

[B77] Kwolek D. Gerstberger S. Tait S. Qiu J. (2023). Ovarian, uterine, and vulvovaginal cancers: Screening, treatment overview, and prognosis. *Med. Clin.* 107 329–355. 10.1016/j.mcna.2022.10.016 36759101

[B78] Kycler W. Kubiak A. Rzymski P. Wilczak M. Trojanowski M. Roszak M. (2017). Impact of selected environmental factors on attendance in the breast and cervical cancer early detection programme in the Wielkopolska province of Poland during 2007-2012. *Ann. Agric. Environ. Med.* 24 467–471. 10.26444/aaem/74481 28954492

[B79] Labani S. Asthana S. Sodhani P. Gupta S. Bhambhani S. Pooja B. (2014). CareHPV cervical cancer screening demonstration in a rural population of north India. *Eur. J. Obstet. Gynecol. Reprod. Biol.* 176 75–79. 10.1016/j.ejogrb.2014.03.006 24685404

[B80] Lasche M. Gallwas J. Grundker C. (2022). Like brothers in arms: How hormonal stimuli and changes in the metabolism signaling cooperate, leading HPV infection to drive the onset of cervical cancer. *Int. J. Mol. Sci.* 23:5050. 10.3390/ijms23095050 35563441 PMC9103757

[B81] Lau K. Hsu Y. Lin Y. Yeap M. Lee C. Chen K. (2021). Case history on Epstein-Barr Virus-associated smooth muscle tumor (EBV-SMT) of cranio-cervical junction in an immunocompetent patient. *Br. J. Neurosurg.* 10.1080/02688697.2021.1932745 [Epub ahead of print]. 34057864

[B82] Lee S. Kang D. Seo S. Jeong J. Yoo K. Jeon Y. (2003). Multiple HPV infection in cervical cancer screened by HPVDNAChip™. *Cancer Lett.* 198 187–192. 10.1016/s0304-3835(03)00312-4 12957357

[B83] Leroux-Roels G. Haelterman E. Maes C. Levy J. De Boever F. Licini L. (2011). Randomized trial of the immunogenicity and safety of the Hepatitis B vaccine given in an accelerated schedule coadministered with the human papillomavirus type 16/18 AS04-adjuvanted cervical cancer vaccine. *Clin. Vaccine Immunol.* 18 1510–1518. 10.1128/CVI.00539-10 21734063 PMC3165228

[B84] Li J. Dai X. Zhang H. Zhang W. Sun S. Gao T. (2015). Up-regulation of human cervical cancer proto-oncogene contributes to hepatitis B virus-induced malignant transformation of hepatocyte by down-regulating E-cadherin. *Oncotarget* 6 29196–29209. 10.18632/oncotarget.5039 26470691 PMC4745720

[B85] Liu X. Ma H. Fei L. Jiang M. Xia M. Bai L. (2020). HPV-mediated down-regulation of NOD1 inhibits apoptosis in cervical cancer. *Infect. Agents Cancer* 15:6. 10.1186/s13027-020-0272-3 32021648 PMC6993450

[B86] Liu Y. Fan P. Yang Y. Xu C. Huang Y. Li D. (2019). Human papillomavirus and human telomerase RNA component gene in cervical cancer progression. *Sci. Rep.* 9:15926. 10.1038/s41598-019-52195-5 PMC6828729 31685833

[B87] Liu Y. Liu H. Sheng B. Pan S. Wang Z. Zhu X. (2022a). The functions of lncRNAs in the HPV-negative cervical cancer compared with HPV-positive cervical cancer. *Apoptosis* 27 685–696. 35980559 10.1007/s10495-022-01761-w

[B88] Liu Y. Zhang Q. Ni R. (2022b). Association between genetic variants (rs920778, rs4759314, and rs217727) in LncRNAs and cervical cancer susceptibility in Chinese population: A systematic review and meta-analysis. *Front. Genet.* 13:988207. 10.3389/fgene.2022.988207 36313463 PMC9608570

[B89] Lok A. McMahon B. (2009). Chronic hepatitis B: Update 2009. *Hepatology* 50 661–662. 19714720 10.1002/hep.23190

[B90] Lourenço de Freitas N. Deberaldini M. Gomes D. Pavan A. Sousa Â Dos Santos J. (2021). Histone deacetylase inhibitors as therapeutic interventions on cervical cancer induced by human papillomavirus. *Front. Cell. Dev. Biol.* 8:592868. 10.3389/fcell.2020.592868 33634093 PMC7901962

[B91] Luo C. Yu S. Zhang J. Wu X. Dou Z. Li Z. (2022). Hepatitis B or C viral infection and the risk of cervical cancer. *Infect. Agents Cancer* 17:54. 10.1186/s13027-022-00466-8 PMC9624004 36320009

[B92] Luo W. Feng Y. Guo R. Tang L. Chen L. Zhou G. (2019). Patterns of EBV-positive cervical lymph node involvement in head and neck cancer and implications for the management of nasopharyngeal carcinoma T0 classification. *Oral Oncol.* 91 7–12. 10.1016/j.oraloncology.2019.01.012 30926066

[B93] Macleod C. Reynolds J. (2021). Human Papilloma Virus infection and cervical cancer among women who sell sex in Eastern and Southern Africa: A scoping review. *Womens Health* 17:17455065211058349. 10.1177/17455065211058349 34775848 PMC8593294

[B94] Mahmood M. Baghestanian M. Thomas W. R. Battistutti W. Pischinger K. Schatten C. (2002). Detection of hepatitis C virus (HCV) RNA in normal cervical smears of HCV-seropositive patients. *Clin. Infect. Dis*. 35, 966–973. 10.1086/342909 12355384

[B95] Malevolti M. Lugo A. Scala M. Gallus S. Gorini G. Lachi A. (2022). Dose-risk relationships between cigarette smoking and cervical cancer: A systematic review and meta-analysis. *Eur. J. Cancer Prev.* 32 171–183. 10.1097/CEJ.0000000000000773 36440802

[B96] Marima R. Hull R. Lolas G. Syrigos K. Kgoebane-Maseko M. Kaufmann A. (2021). The catastrophic HPV/HIV dual viral oncogenomics in concert with dysregulated alternative splicing in cervical cancer. *Int. J. Mol. Sci.* 22:10115. 10.3390/ijms221810115 34576278 PMC8472041

[B97] Markowitz L. Tsu V. Deeks S. Cubie H. Wang S. Vicari A. (2012). Human papillomavirus vaccine introduction–the first five years. *Vaccine* 30 F139–F148. 23199957 10.1016/j.vaccine.2012.05.039

[B98] Marlow L. Waller J. Wardle J. (2007). Public awareness that HPV is a risk factor for cervical cancer. *Br. J. Cancer* 97 691–694. 17687335 10.1038/sj.bjc.6603927 PMC2360359

[B99] Maskey N. Thapa N. Maharjan M. Shrestha G. Maharjan N. Cai H. (2019). Infiltrating CD4 and CD8 lymphocytes in HPV infected uterine cervical milieu. *Cancer Manag. Res.* 11 7647–7655. 31616181 10.2147/CMAR.S217264 PMC6698604

[B100] Moharreri M. Sohrabi A. (2021). Characteristics of hsv-2, *M. genitalium* and *C. trachomatis* in HPV genotypes associated with cervical intraepithelial neoplasia and genital infections. *Infect. Disord. Drug Targets* 21 112–118. 10.2174/1871526520666191231142317 31889502

[B101] Montoya-Fuentes H. Suárez Rincón A. Ramírez-Muñoz M. Arévalo-Lagunas I. Morán Moguel M. Gallegos Arreola M. (2001). The detection of human papillomavirus 16, 18, 35 and 58 in cervical-uterine cancer and advanced degree of squamous intraepithelial lesions in Western Mexico: Clinical-molecular correlation. *Ginecol. Obstet. Mexico* 69 137–142. 11452411

[B102] Moscicki A. Flowers L. Huchko M. Long M. MacLaughlin K. Murphy J. (2019). Guidelines for cervical cancer screening in immunosuppressed women without HIV infection. *J. Low. Genit. Tract Dis.* 23 87–101. 30907775 10.1097/LGT.0000000000000468

[B103] Mukerebe C. (2022). Kaposi’s sarcoma-associated herpesvirus shedding in saliva and cervical secretions in Tanzanian women. *Tanzania J. Health Res.* 23 157–158. 10.1093/ofid/ofae161 PMC11036159 38654970

[B104] Muñoz N. Bosch F. de Sanjosé S. Herrero R. Castellsagué X. Shah K. (2003). Epidemiologic classification of human papillomavirus types associated with cervical cancer. *N. Engl. J. Med.* 348 518–527. 12571259 10.1056/NEJMoa021641

[B105] Muñoz-Bello J. Carrillo-García A. Lizano M. (2022). Epidemiology and molecular biology of HPV variants in cervical cancer: The state of the art in Mexico. *Int. J. Mol. Sci.* 23:8566. 10.3390/ijms23158566 35955700 PMC9368912

[B106] Nakagawa J. Schirmer J. Barbieri M. (2010). Human papillomavirus (HPV) and uterine cervical cancer. *Rev. Bras. Enferm.* 63 307–311. 20521005 10.1590/s0034-71672010000200021

[B107] Nelson E. Maynard B. Loux T. Fatla J. Gordon R. Arnold L. (2017). The acceptability of self-sampled screening for HPV DNA: A systematic review and meta-analysis. *Sex. Transm. Infect.* 93 56–61. 10.1136/sextrans-2016-052609 28100761

[B108] Noel J. Lespagnard L. Fayt I. Verhest A. Dargent J. (2001). Evidence of human papilloma virus infection but lack of Epstein-Barr virus in lymphoepithelioma-like carcinoma of uterine cervix: Report of two cases and review of the literature. *Hum. Pathol.* 32 135–138. 10.1053/hupa.2001.20901 11172309

[B109] Ogilvie G. Krajden M. Maginley J. Isaac-Renton J. Hislop G. Elwood-Martin R. (2007). Feasibility of self-collection of specimens for human papillomavirus testing in hard-to-reach women. *CMAJ* 177 480–483. 17724326 10.1503/cmaj.070013 PMC1950166

[B110] Okoye J. Ngokere A. Onyenekwe C. Eze U. Obioma O. (2023). Abstract C029: The pattern of mutant p53 protein expression in cervical cancer-associated single and co-infection with human papillomavirus and Epstein-Barr virus. *Cancer Epidemiol. Biomarkers Prev.* 32:C029.

[B111] Pandey N. Chauhan A. Raithatha N. Patel P. Khandelwal R. Desai A. (2021). Influence of TLR4 and TLR9 polymorphisms and haplotypes on multiple hrHPV infections and HPV16 copy number in cervical cancer and cervicitis. *Microb. Pathog.* 159:105149. 10.1016/j.micpath.2021.105149 34416273

[B112] Petignat P. Faltin D. Bruchim I. Tramèr M. Franco E. Coutlée F. (2007). Are self-collected samples comparable to physician-collected cervical specimens for human papillomavirus DNA testing? A systematic review and meta-analysis. *Gynecol. Oncol.* 105 530–535. 17335880 10.1016/j.ygyno.2007.01.023

[B113] Pham C. Juhasz M. Sung C. Mesinkovska N. (2020). The human papillomavirus vaccine as a treatment for human papillomavirus–related dysplastic and neoplastic conditions: A literature review. *J. Am. Acad. Dermatol.* 82 202–212. 31085272 10.1016/j.jaad.2019.04.067

[B114] Plummer M. de Martel C. Vignat J. Ferlay J. Bray F. Franceschi S. (2016). Global burden of cancers attributable to infections in 2012: A synthetic analysis. *Lancet Glob. Health* 4 e609–e616. 27470177 10.1016/S2214-109X(16)30143-7

[B115] Porter V. Marra M. (2022). The drivers, mechanisms, and consequences of genome instability in HPV-driven cancers. *Cancers* 14:4623. 10.3390/cancers14194623 36230545 PMC9564061

[B116] Racey C. Withrow D. Gesink D. (2013). Self-collected HPV testing improves participation in cervical cancer screening: A systematic review and meta-analysis. *Can. J. Public Health* 104 e159–e166. 10.1007/bf03405681 23618210 PMC6973997

[B117] Ronco G. Dillner J. Elfström K. Tunesi S. Snijders P. Arbyn M. (2014). Efficacy of HPV-based screening for prevention of invasive cervical cancer: Follow-up of four European randomised controlled trials. *Lancet* 383 524–532. 10.1016/S0140-6736(13)62218-7 24192252

[B118] Rosadas C. Taylor G. (2022). HTLV-1 and co-infections. *Front. Med.* 9:812016. 10.3389/fmed.2022.812016 35187000 PMC8850362

[B119] Roy V. Jung W. Linde C. Coates E. Ledgerwood J. Costner P. (2023). Differences in HPV-specific antibody Fc-effector functions following Gardasil^®^ and Cervarix^®^ vaccination. *Npj Vaccines* 8:39. 10.1038/s41541-023-00628-8 36922512 PMC10017795

[B120] Safaeian M. Solomon D. Castle P. (2007). Cervical cancer prevention—cervical screening: Science in evolution. *Obstet. Gynecol. Clin. North Am.* 34 739–760. 10.1016/j.ogc.2007.09.004 18061867 PMC2762353

[B121] Sancho-Garnier H. Tamalet C. Halfon P. Leandri F. Le Retraite L. Djoufelkit K. (2013). HPV self-sampling or the Pap-smear: A randomized study among cervical screening nonattenders from lower socioeconomic groups in France. *Int. J. Cancer* 133 2681–2687. 10.1002/ijc.28283 23712523

[B122] Sankaranarayanan R. Nene B. Shastri S. Jayant K. Muwonge R. Budukh A. (2009). HPV screening for cervical cancer in rural India. *N. Engl. J. Med.* 360 1385–1394. 19339719 10.1056/NEJMoa0808516

[B123] Sasagawa T. Shimakage M. Nakamura M. Sakaike J. Ishikawa H. Inoue M. (2000). Epstein-Barr virus (EBV) genes expression in cervical intraepithelial neoplasia and invasive cervical cancer: A comparative study with human papillomavirus (HPV) infection. *Hum. Pathol.* 31 318–326. 10.1016/s0046-8177(00)80245-2 10746674

[B124] Sassenou J. Ringa V. Zins M. Ozguler A. Paquet S. Panjo H. (2021). Women with obesity in cervical cancer screening. The double penalty: Underscreening and income inequalities. *Obes. Res. Clin. Pract.* 15 212–215. 10.1016/j.orcp.2021.03.003 33771444

[B125] Schierhout G. McGregor S. Gessain A. Einsiedel L. Martinello M. Kaldor J. (2020). Association between HTLV-1 infection and adverse health outcomes: A systematic review and meta-analysis of epidemiological studies. *Lancet Infect. Dis.* 20 133–143. 10.1016/S1473-3099(19)30402-5 31648940

[B126] Schiffman M. Wentzensen N. Wacholder S. Kinney W. Gage J. Castle P. (2011). Human papillomavirus testing in the prevention of cervical cancer. *J. Natl. Cancer Inst.* 103 368–383. 21282563 10.1093/jnci/djq562 PMC3046952

[B127] Serrano B. Ibáñez R. Robles C. Peremiquel-Trillas P. de Sanjosé S. Bruni L. (2022). Worldwide use of HPV self-sampling for cervical cancer screening. *Prev. Med.* 154:106900. 10.1016/j.ypmed.2021.106900 34861338

[B128] Shiels M. Engels E. Linet M. Clarke C. Li J. Hall H. (2013). The epidemic of non–hodgkin lymphoma in the United States: Disentangling the effect of HIV, 1992–2009U. S. NHL trends excluding HIV-infected cases, 1992–2009. *Cancer Epidemiol. Biomarkers Prev.* 22 1069–1078. 23595542 10.1158/1055-9965.EPI-13-0040 PMC3698875

[B129] Shin S. Carpenter C. Ekstrand M. Wang Q. Grover S. Zetola N. (2019). Cervical cancer awareness and presence of abnormal cytology among HIV-infected women on antiretroviral therapy in rural Andhra Pradesh, India. *Int. J. STD AIDS* 30 586–595. 10.1177/0956462419825950 30813859 PMC6510620

[B130] Singh G. Sharma S. Singh S. (2022). miR-34a negatively regulates cell cycle factor Cdt2/DTL in HPV infected cervical cancer cells. *BMC Cancer* 22:777. 10.1186/s12885-022-09879-5 35840896 PMC9288023

[B131] Siu S. Cheung T. Chan P. Lin C. Lo K. (2007). Patients with malignant or pre-malignant cervical lesion have increased risk of becoming hepatitis B carrier. *J. Exp. Clin. Cancer Res.* 26 77–81. 17550135

[B132] Smith J. Vaz O. Gaber C. Des Marais A. Chirumamilla B. Hendrickson L. (2023). Recruitment strategies and HPV self-collection return rates for under-screened women for cervical cancer prevention. *PLoS One* 18:e0280638. 10.1371/journal.pone.0280638 36952486 PMC10035812

[B133] Sosse S. Tadlaoui K. Benhassou M. Elkarroumi M. Elmzibri M. Ennaji M. (2022). Viral co-infection of oncogenic human papillomavirus with Epstein–Barr Virus, human herpesvirus 8 and Herpes Simplex Virus type 2 in malignant cervical cancer. *Int. Med. J.* 30.

[B134] Srinath A. van Merode F. Rao S. Pavlova M. (2022). Barriers to cervical cancer and breast cancer screening uptake in low-and-middle-income countries: A systematic review. *Health Policy Plan* 38 509–527. 10.1093/heapol/czac104 36525529 PMC10089064

[B135] Stelzle D. Tanaka L. Lee K. Ibrahim Khalil A. Baussano I. Shah A. (2021). Estimates of the global burden of cervical cancer associated with HIV. *Lancet Glob. Health* 9 e161–e169. 33212031 10.1016/S2214-109X(20)30459-9 PMC7815633

[B136] Tawe L. MacDuffie E. Narasimhamurthy M. Wang Q. Gaseitsiwe S. Moyo S. (2020). Human papillomavirus genotypes in women with invasive cervical cancer with and without human immunodeficiency virus infection in Botswana. *Int. J. Cancer* 146 1667–1673. 31325316 10.1002/ijc.32581 PMC7055961

[B137] Tin K. Ngamjarus C. Rattanakanokchai S. Sothornwit J. Aue-Aungkul A. Paing A. (2023). Interventions to increase the uptake of cervical cancer screening in low- and middle-income countries: A systematic review and meta-analysis. *BMC Womens Health* 23:120. 10.1186/s12905-023-02265-8 36959632 PMC10035175

[B138] Ton M. Swami N. Germar M. Dee E. (2022). HPV mRNA testing in cervical cancer screening: Implications for low- and middle-income countries. *Int. J. Gynecol. Cancer* 32 1632–1633. 10.1136/ijgc-2022-003959 36220194 PMC9822850

[B139] Tota J. Ramanakumar A. Jiang M. Dillner J. Walter S. Kaufman J. (2013). Epidemiologic approaches to evaluating the potential for human papillomavirus type replacement postvaccination. *Am. J. Epidemiol.* 178 625–634. 10.1093/aje/kwt018 23660798 PMC3736757

[B140] Urbute A. Kjaer S. Kesmodel U. Frederiksen K. Thomsen L. (2022). Women with obesity participate less in cervical cancer screening and are more likely to have unsatisfactory smears: Results from a nationwide Danish cohort study. *Prev. Med.* 159:107072. 10.1016/j.ypmed.2022.107072 35460722

[B141] van den Helder R. Steenbergen R. van Splunter A. Mom C. Tjiong M. Martin I. (2022). HPV and DNA methylation testing in urine for cervical intraepithelial neoplasia and cervical cancer detection. *Clin. Cancer Res.* 28 2061–2068. 35266975 10.1158/1078-0432.CCR-21-3710

[B142] Vitali D. Bagri P. Wessels J. Arora M. Ganugula R. Parikh A. (2020). Curcumin can decrease tissue inflammation and the severity of HSV-2 infection in the female reproductive mucosa. *Int. J. Mol. Sci.* 21:337. 10.3390/ijms21010337 31947962 PMC6982333

[B143] Vranic S. Cyprian F. Akhtar S. Al Moustafa A. (2018). The role of Epstein-Barr Virus in cervical cancer: A brief update. *Front. Oncol.* 8:113. 10.3389/fonc.2018.00113 29719817 PMC5913353

[B144] Walboomers J. M. Jacobs M. V. Manos M. M. Bosch F. X. Kummer J. A. Shah K. V. (1999). Human papillomavirus is a necessary cause of invasive cervical cancer worldwide. *J. Pathol.* 189 12–19. 10451482 10.1002/(SICI)1096-9896(199909)189:1<12::AID-PATH431>3.0.CO;2-F

[B145] Waller J. Bartoszek M. Marlow L. Wardle J. (2009). Barriers to cervical cancer screening attendance in England: A population-based survey. *J. Med. Screen.* 16 199–204. 10.1258/jms.2009.009073 20054095

[B146] Wu X. Li L. Li Y. Jiang M. Li K. Li Z. (2021). Prognostic value of serological markers of hepatitis B virus infection in squamous cell cervical cancer. *J. Cancer* 12 6620–6628. 10.7150/jca.61249 34659552 PMC8518014

[B147] Yadav C. Yadav R. Chhabra R. Nanda S. Ranga S. Kadian L. (2023a). Correction to: Overview of genetic and epigenetic regulation of human papillomavirus and apoptosis in cervical cancer. *Apoptosis* 10.1007/s10495-023-01822-8 [Epub ahead of print]. 36757581

[B148] Yadav C. Yadav R. Chabbra R. Nanda S. Ranga S. Kadian L. (2023b). Overview of genetic and epigenetic regulation of human papillomavirus and apoptosis in cervical cancer. *Apoptosis* 10.1007/s10495-023-01812-w [Epub ahead of print]. 36652131

[B149] Yordanov A. Ivanov I. Dineva T. Popovska S. Karcheva M. Strashilov S. (2020). Lymphoepithelioma-like carcinoma of the uterine cervix: Correlation with Epstein-Barr virus and human papillomavirus infection. A single-center experience. *Eur. J. Gynaecol. Oncol*. 41, 913–918. 10.31083/j.ejgo.2020.06.2107 31345004

[B150] Yordanov A. Karcheva M. Betova T. Ivanov I. Dineva T. Strashilov S. (2019). Immunohistochemical study of human papilloma virus and Epstein–Barr virus in patients with lymphoepithelioma-like carcinoma of the uterine cervix. *Arch. Balk Med. Union* 53 680–684.

[B151] Youn J. Hur S. Woo J. Kim Y. Lim M. Park S. (2020). Pembrolizumab plus GX-188E therapeutic DNA vaccine in patients with HPV-16-positive or HPV-18-positive advanced cervical cancer: Interim results of a single-arm, phase 2 trial. *Lancet Oncol.* 21 1653–1660. 10.1016/S1470-2045(20)30486-1 33271094

[B152] Yuan M. Zhao X. Wang H. Hu S. Zhao F. (2023). Trend in cervical cancer incidence and mortality rates in China, 2006-2030: A Bayesian age-period-cohort modeling study. *Cancer Epidemiol. Biomarkers Prev.* 10.1158/1055-9965.EPI-22-0674 [Epub ahead of print]. 36944168

[B153] Zhang J. Yu G. Yang Y. Wang Y. Guo M. Yin Q. (2022). A small-molecule inhibitor of MDMX suppresses cervical cancer cells via the inhibition of E6-E6AP-p53 axis. *Pharmacol. Res.* 177:106128. 10.1016/j.phrs.2022.106128 35150860

[B154] Zidi S. Sahli M. Mezlini A. Yacoubli-Loueslati B. (2020). Association of combined tobacco smoking, hormonal contraceptive use and status matrimonial with cervical cancer evolution in Tunisian women. *Pathol. Oncol. Res.* 26 217–222. 10.1007/s12253-018-0442-4 29872962

[B155] zur Hausen H. (2002). Papillomaviruses and cancer: From basic studies to clinical application. *Nat. Rev. Cancer* 2 342–350. 12044010 10.1038/nrc798

